# Performance of a Large Language Model in Screening Citations

**DOI:** 10.1001/jamanetworkopen.2024.20496

**Published:** 2024-07-08

**Authors:** Takehiko Oami, Yohei Okada, Taka-aki Nakada

**Affiliations:** 1 Department of Emergency and Critical Care Medicine, Chiba University Graduate School of Medicine, Chiba, Japan; 2 Department of Preventive Services, Kyoto University Graduate School of Medicine, Kyoto, Japan; 3 Health Services and Systems Research, Duke-NUS Medical School, National University of Singapore, Singapore

## Abstract

**Importance:**

Large language models (LLMs) are promising as tools for citation screening in systematic reviews. However, their applicability has not yet been determined.

**Objective:**

To evaluate the accuracy and efficiency of an LLM in title and abstract literature screening.

**Design, Setting, and Participants:**

This prospective diagnostic study used the data from the title and abstract screening process for 5 clinical questions (CQs) in the development of the Japanese Clinical Practice Guidelines for Management of Sepsis and Septic Shock. The LLM decided to include or exclude citations based on the inclusion and exclusion criteria in terms of patient, population, problem; intervention; comparison; and study design of the selected CQ and was compared with the conventional method for title and abstract screening. This study was conducted from January 7 to 15, 2024.

**Exposures:**

LLM (GPT-4 Turbo)–assisted citation screening or the conventional method.

**Main Outcomes and Measures:**

The sensitivity and specificity of the LLM-assisted screening process was calculated, and the full-text screening result using the conventional method was set as the reference standard in the primary analysis. Pooled sensitivity and specificity were also estimated, and screening times of the 2 methods were compared.

**Results:**

In the conventional citation screening process, 8 of 5634 publications in CQ 1, 4 of 3418 in CQ 2, 4 of 1038 in CQ 3, 17 of 4326 in CQ 4, and 8 of 2253 in CQ 5 were selected. In the primary analysis of 5 CQs, LLM-assisted citation screening demonstrated an integrated sensitivity of 0.75 (95% CI, 0.43 to 0.92) and specificity of 0.99 (95% CI, 0.99 to 0.99). Post hoc modifications to the command prompt improved the integrated sensitivity to 0.91 (95% CI, 0.77 to 0.97) without substantially compromising specificity (0.98 [95% CI, 0.96 to 0.99]). Additionally, LLM-assisted screening was associated with reduced time for processing 100 studies (1.3 minutes vs 17.2 minutes for conventional screening methods; mean difference, −15.25 minutes [95% CI, −17.70 to −12.79 minutes]).

**Conclusions and Relevance:**

In this prospective diagnostic study investigating the performance of LLM-assisted citation screening, the model demonstrated acceptable sensitivity and reasonably high specificity with reduced processing time. This novel method could potentially enhance efficiency and reduce workload in systematic reviews.

## Key Points

**Question:**

How accurate and efficient is a large language model (LLM) for screening titles and abstracts for article inclusion in a systematic review?

**Findings:**

In this diagnostic study, LLM-assisted citation screening exhibited acceptable sensitivity and reasonably high specificity in evaluating 5 clinical questions, with post hoc prompt modifications further improving accuracy. The screening time for 100 studies was significantly reduced compared with that of conventional methods.

**Meaning:**

These findings suggest that LLM-assisted citation screening could offer a reliable and time-efficient alternative to systematic review processes.

## Teaser

This diagnostic study examines how a large language model used for title and abstract screening for systematic reviews compares in accuracy and efficiency with conventional screening methods.

## Introduction

Clinical practice guidelines are systematically developed based on a synthesis of the current best evidence and provide clinicians and patients with essential guidance for clinical decision-making. During guideline development, collecting and compiling the latest research findings in systematic reviews is a critical process requiring extensive work and effort for screening the relevant literature, thereby presenting a major challenge in the development of such guidelines.^1,2,3^ Recent progress in applying machine learning to streamline this process can potentially reduce the effort.^4,5,6,7,8,9^ However, our previous research on the use of machine learning^10^ indicated that while time efficiency improved, the precision of the results failed to reach the desired level of accuracy, with a sensitivity and specificity of 0.24 to 0.80 and 0.99 to 1.00, respectively. Accordingly, a more precise method for citation screening in systematic reviews requires further investigation.^9,10,11^

Along with the growing interest in large language models (LLMs), these advanced artificial intelligence tools have showcased the capacity to perform complicated tasks, such as data analysis and text generation using natural language processing techniques.^12,13,14,15^ Although previous reports have suggested the feasibility of harnessing an LLM for citation screening tasks,^16,17^ studies on the deployment of an LLM for extensive citation screening in the development of clinical practice guidelines remain lacking.

We hypothesized that LLM-assisted citation screening can potentially achieve the quality of manual citation screening and significantly reduce the required manual workload. Thus, in this prospective study, we aimed to critically evaluate the accuracy and operational efficiency of LLM-assisted citation screening compared with those of conventional screening methods using clinical questions (CQs) from the Japanese Clinical Practice Guidelines for the Management of Sepsis and Septic Shock (J-SSCG).

## Methods

### Study Design

We conducted a prospective diagnostic study to evaluate the accuracy of citation screening using LLMs. To ensure transparency and reproducibility, we submitted our research protocol to the *medRxiv* preprint server and the University Hospital Medical Information Network (UMIN) Clinical Trials Registry under the identifier UMIN000053091 on December 31, 2023.^18^ We did not seek institutional review board approval because the study did not meet the definition of human participant research. We followed the Standards for Reporting of Diagnostic Accuracy (STARD) guidelines.

### Setting

We evaluated the accuracy and efficiency of the LLM-assisted citation screening method using data from the title and abstract screening process for 5 CQs in the J-SSCG 2024. The CQs are described in eTable 1 in Supplement 1. Details of the J-SSCG 2024 development process have been described in a previous report,^10^ and the conventional process described later was completed when this study was being conducted (eAppendix in Supplement 1).

### Conventional Citation Screening

Through the conventional citation screening process, literature was selected by 2 independent reviewers who were clinical experts. The details of the conventional citation screening are described in the eAppendix in Supplement 1.

### LLM Screening

We used GPT-4 Turbo (OpenAI), released on November 7, 2023, as an LLM to evaluate the accuracy and efficiency of citation screening in the development of clinical practice guidelines. To develop the LLM-assisted citation screening, we formulated a query for the LLM according to the guidelines of prompt engineering.^19,20^ We also established a command to enable the LLM to autonomously perform citation screening using pandas (version 1.0.5) in Python (version 3.9.0) via the application programming interface. Each command included a request to the LLM to automatically implement a citation screening task according to the inclusion and exclusion criteria based on the exact wording of the patient, population, problem; intervention; comparison; and study design (PICO) sheet in each CQ described by the J-SSCG 2024 committee in conventional citation screening processes (eTable 1 and eFigure 1 in Supplement 1). After importing the 5 sets of literature used in conventional citation screening, the LLM, without prior knowledge, decided to include or exclude each citation based on the inclusion and exclusion criteria in terms of the PICO sheet of the selected CQ. To assess the workload, we recorded the processing time required to complete the task using Python. The LLM-assisted screening process was performed from January 7 to 15, 2024, in English. The code for this process is available online.^21^

### Statistical Analysis

We evaluated the accuracy of the LLM-assisted citation screening by calculating the sensitivity and specificity with a 95% CI. In the primary analysis, we used results of the full-text screening session with the conventional method as the reference standard because these publications were included in the qualitative evaluation. In the secondary analysis, results of the title and abstract screening session using the conventional method were used as the reference standards. In addition to the primary and secondary analysis, using meta-analysis techniques, we also calculated the pooled sensitivity and specificity as overall values for the results in primary, secondary, and post hoc analyses described later, in accordance with the Cochrane Handbook.^22^ We applied a random-effects model to account for both within-study and between-study variance.^23^ We evaluated heterogeneity of the CQs using the χ^2^ test with inconsistency values (*I*^2^).

Duration of the citation screening process for each CQ was compared for the 2 methods. A summary of continuous variables is presented as either mean with SD or median with IQR, as appropriate. Based on the normality of the distribution, the unpaired *t* test was used for statistical analysis. The meta package for the meta-analysis in R version 4.1.2 (R Foundation for Statistical Computing) was used, and all other statistical analyses were performed using the Prism version 9 software (GraphPad Software).

As a post hoc analysis, we reviewed the LLM results in cases of false-positive and/or false-negative results to explore why the LLM incorrectly judged the result. We used this review as a basis to modify the prompt to optimize the accuracy of the LLM and examine how prompt modification affected the LLM’s performance on citation screening tasks. We also conducted another post hoc analysis involving 3 iterations of LLM querying and adopted a majority-voting strategy to address the variability and enhance the robustness of LLM-assisted screening.^24^ In this analysis, we included studies that were deemed relevant to any of the 3 LLM-assisted screenings. Furthermore, we incorporated the chain-of -thought strategy into the modified prompt.^25^ Additionally, to assess the outcomes of LLM-based methods on the results of the meta-analyses followed by the citation screening, we incorporated a post hoc meta-analysis that utilized studies selected through LLM-based methods, comparing these results with those obtained using conventional methods, where available. These post hoc analyses were conducted from January 17 to 19, 2024, and from April 13 to 19, 2024.

## Results

### Conventional Citation Screening

**Figure 1.**
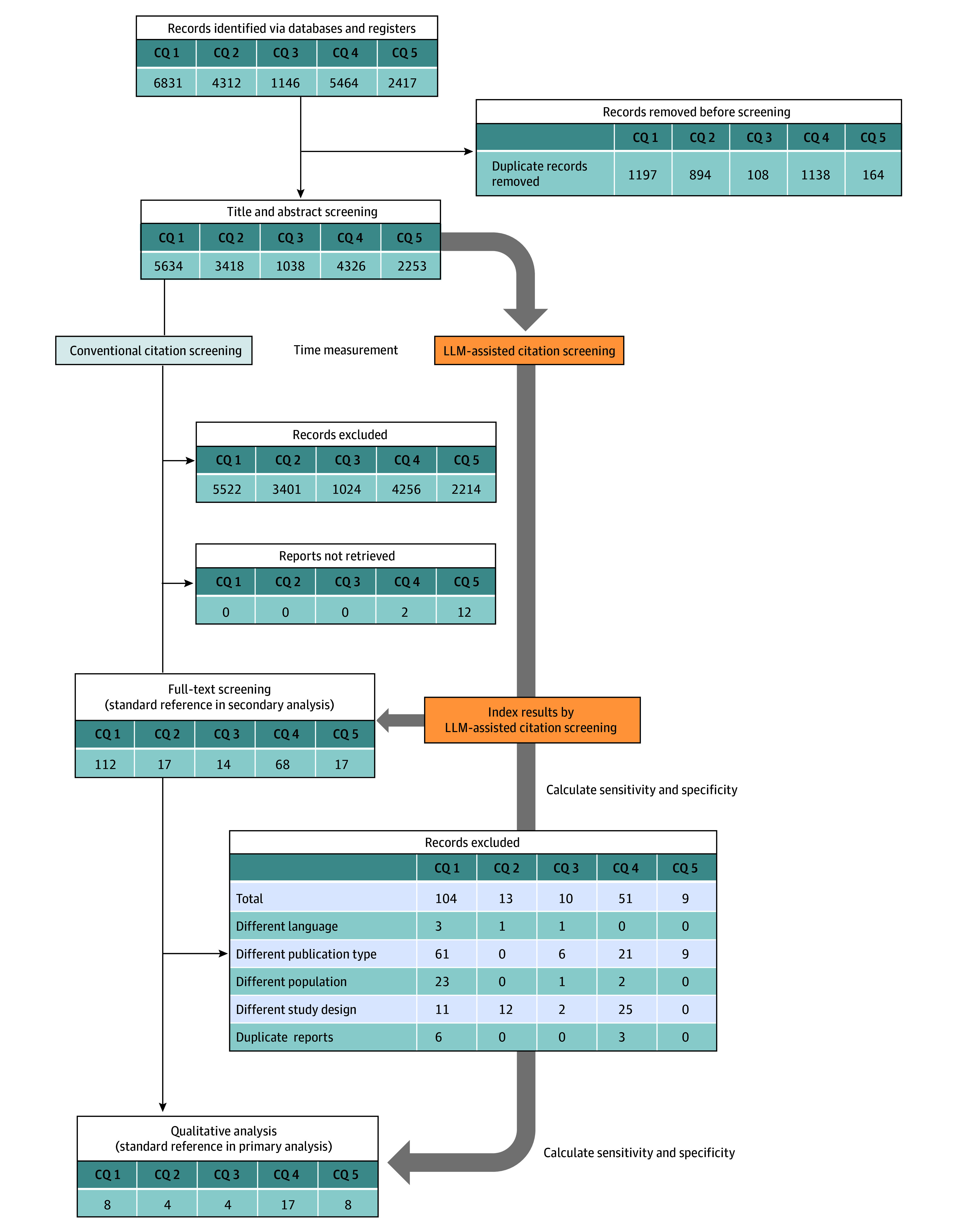
Schematic Diagram of Systematic Review Using Large Language Model (LLM)–Assisted Citation Screening and the Conventional Method Flowchart of the systematic review through identification, title and abstract screening, and full-text screening. Timing of the statistics on the accuracy and measurement of the screening time between LLM-assisted screening and conventional method in the primary and secondary analyses are also depicted. CQ indicates clinical question.

During the conventional citation screening process, 112 of 5634 publications in CQ 1 (2.0%), 17 of 3418 in CQ 2 (0.5%), 14 of 1038 in CQ 3 (1.3%), 70 of 4326 in CQ 4 (1.6%), and 39 of 2253 in CQ 5 (1.7%) were selected in the title and abstract screening session. A total of 41 publications, including 8 for CQ 1, 4 for CQ 2, 4 for CQ 3, 17 for CQ 4, and 8 for CQ 5, were selected for qualitative analysis in the full-text screening session within each systematic review (Figure 1).

### Primary Analysis of the Accuracy of LLM-Assisted Citation Screening

**Figure 2.**
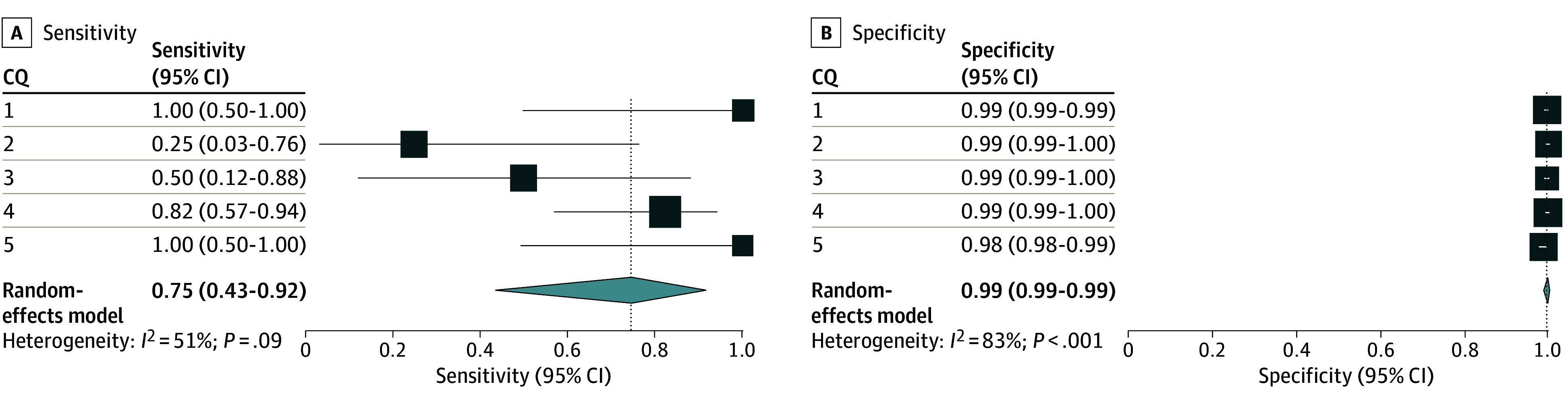
Accuracy of Large Language Model–Assisted Citation Screening in the Primary Analysis The primary analysis used results of the included publications for qualitative analysis, using the conventional method as the standard reference. The individual sensitivity and specificity results for each clinical question (CQ) and integrated sensitivities and specificities across CQ 1 to 5 are shown, with confidence intervals and inconsistency values (*I*^2^).

In LLM-assisted citation screening, 8 publications for CQ 1, 1 for CQ 2, 2 for CQ 3, 14 for CQ 4, and 8 for CQ 5 were included in the qualitative analysis (eTable 2 in Supplement 1). In the primary analysis, the sensitivity and specificity of the index results of LLM-assisted screening were 1.00 (95% CI, 0.50-1.00) and 0.99 (95% CI, 0.99-0.99) for CQ 1, 0.25 (95% CI, 0.03-0.76) and 0.99 (95% CI, 0.99-1.00) for CQ 2, 0.50 (95% CI, 0.12-0.88) and 0.99 (95% CI, 0.99-1.00) for CQ 3, 0.82 (95% CI, 0.57-0.94) and 0.99 (95% CI, 0.99-1.00) for CQ 4, and 1.00 (95% CI, 0.50-1.00) and 0.98 (95% CI, 0.98-0.99) for CQ 5, respectively (Figure 2). The numbers of true-positive, true-negative, false-positive, and false-negative results are listed in eTable 2 in Supplement 1. Meta-analysis showed that the integrated sensitivity and specificity values among the 5 CQs were 0.75 (95% CI, 0.43-0.92) and 0.99 (95% CI, 0.99-0.99), respectively (Figure 2).

### Secondary Analysis of the Accuracy of LLM-Assisted Citation Screening

**Figure 3.**
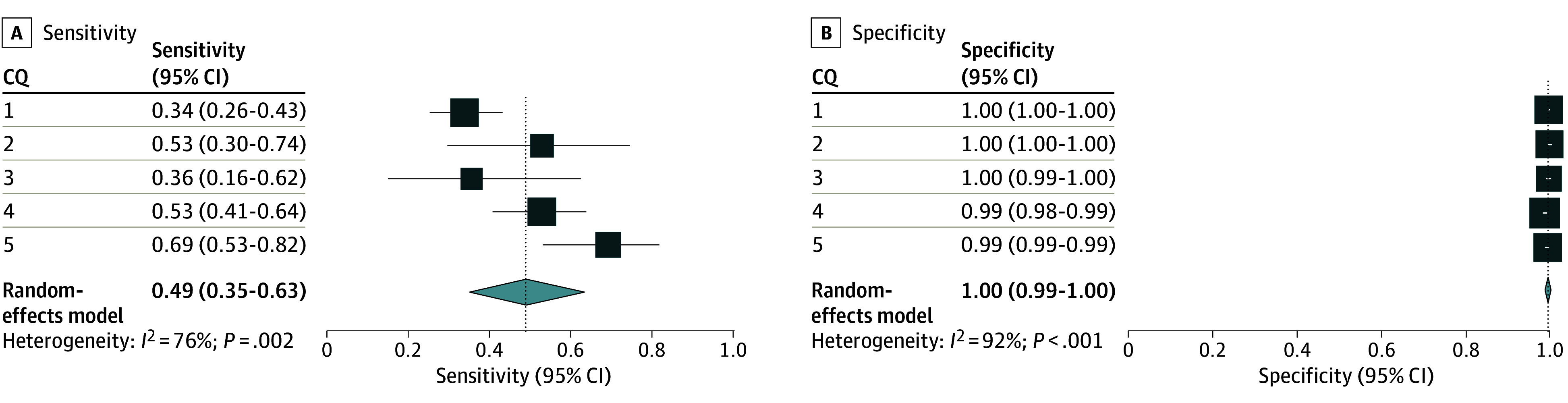
Accuracy of Large Language Model–Assisted Citation Screening in the Secondary Analysis Secondary analysis used results of the included publications for the full-text screening session using the conventional method as the standard reference. The individual sensitivities and specificities for each clinical question (CQ) and integrated sensitivities across CQ 1 to 5 are shown, with confidence intervals and inconsistency values (*I*^2^).

In the secondary analysis, the integrated sensitivity and specificity values across the 5 CQs were 0.49 (95% CI, 0.35-0.63) and 1.00 (95% CI, 0.99-1.00), respectively (Figure 3; eAppendix in Supplement 1). The numbers of true-positive, true-negative, false-positive, and false-negative results are listed in eTable 2 in Supplement 1.

### Comparison of Overall Citation Screening Time for 100 Studies Between the LLM-Assisted and Conventional Methods

The LLM-assisted screening method resulted in significantly shorter overall processing time for 100 studies (1.30 [95% CI, 1.28-1.32] minutes) compared with the conventional screening method (17.2 [95% CI, 14.2–18.6] minutes) (unpaired *t* test: mean difference, −15.25 minutes; 95% CI, −17.70 to −12.79 minutes; *P* < .001) (eAppendix, eTable 3, and eFigure 2 in Supplement 1).

### Post Hoc Analysis Using the Modified Prompt

**Figure 4.**
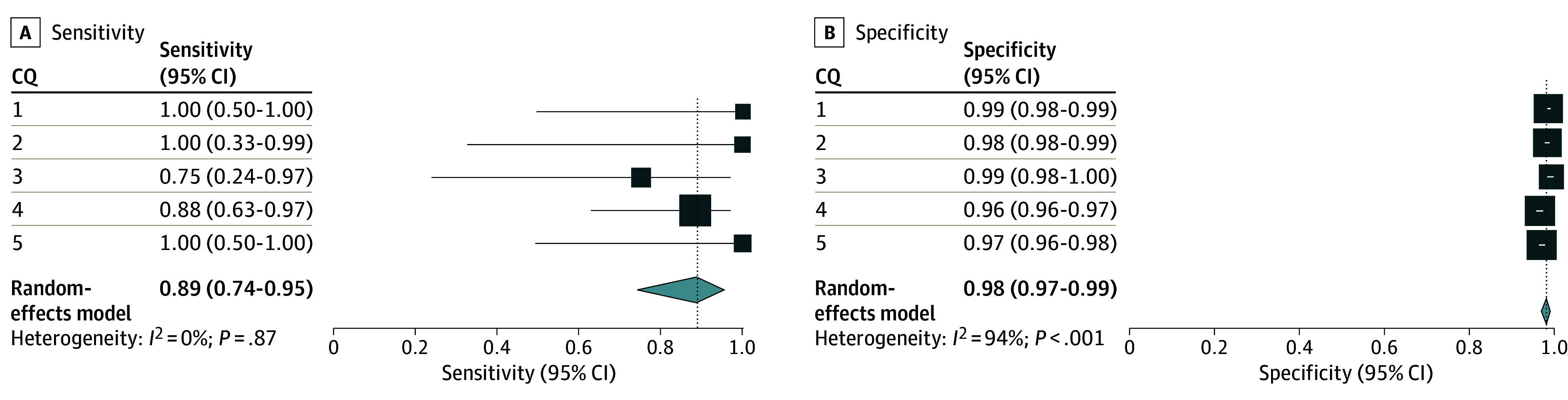
Post Hoc Analysis for the Primary Analysis Using the Modified Prompt Post hoc primary analysis adopted a modified prompt based on false-negative studies. The results of the included publications for qualitative analysis using conventional methods were used as the standard reference. The individual sensitivities and specificities for each clinical question (CQ) and the integrated sensitivities across CQ 1 to 5 are shown with confidence intervals and inconsistency values (*I*^2^).

In the post hoc analysis using the modified command (eAppendix, eTable 4, and eFigure 3 in Supplement 1), the integrated sensitivity and specificity values among the 5 CQs were 0.89 (95% CI, 0.74-0.95) and 0.98 (95% CI, 0.97-0.99), respectively (Figure 4). The numbers of true-positive, true-negative, false-positive, and false-negative results are listed in eTable 5A in Supplement 1.

### Post Hoc Analysis Using Majority-Vote and Chain-of-Thought Strategies

**Figure 5.**
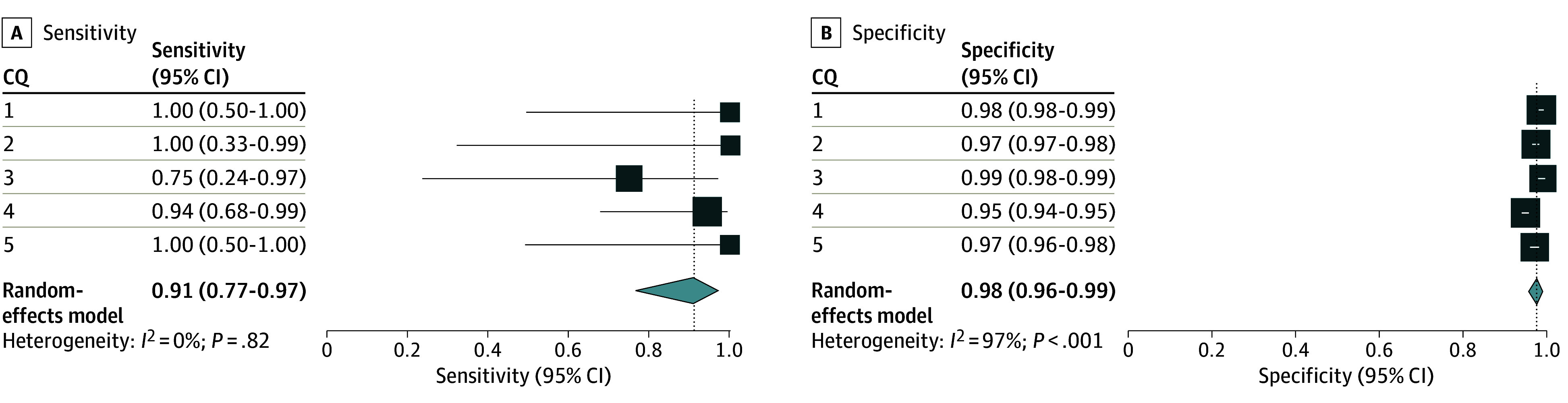
Post Hoc Analysis for the Primary Analysis Using a Majority-Vote Strategy and the Modified Prompt Post hoc primary analysis adopted a majority-vote strategy using a modified prompt based on false-negative studies. The results of the included publications for qualitative analysis using conventional methods were used as the standard reference. The individual sensitivities and specificities for each clinical question (CQ) and the integrated sensitivities across CQ 1 to 5 are shown with confidence intervals and inconsistency values (*I*^2^).

With the original prompt and a majority-vote strategy, the integrated sensitivity and specificity values among the 5 CQs were 0.75 (95% CI, 0.43-0.92) and 0.99 (95% CI, 0.98-0.99) in the primary analysis (eFigure 5 and eTable 5B in Supplement 1). Using the modified prompt and a majority vote, the aggregate sensitivity and specificity values among the 5 CQs were 0.91 (95% CI, 0.77-0.97) and 0.98 (95% CI, 0.96-0.99) in the primary analysis (Figure 5; eTable 5C in Supplement 1).

With the original prompt and the chain-of-thought strategy (eFigure 8 in Supplement 1), the integrated sensitivity and specificity values among the 5 CQs were 0.71 (95% CI, 0.45-0.88) and 0.99 (95% CI, 0.98-0.99) in the primary analysis (eFigure 9 and eTable 5D in Supplement 1). Using the modified prompt and the chain-of-thought strategy, the aggregate sensitivity and specificity values among the 5 CQs were 0.87 (95% CI, 0.67-0.96) and 0.98 (95% CI, 0.96-0.99) in the primary analysis (eFigure 11 and eTable 5E in Supplement 1). The results of post hoc analysis for the secondary analysis are shown in eTable 5, eFigure 4, eFigure 6, eFigure 7, eFigure 10, and eFigure 12 in Supplement 1.

### Association of LLM-Assisted Citation Screening With Results of the Meta-Analysis

The results of the meta-analysis were comparable between the 2 screening methods. This finding indicates that studies classified as false negatives did not substantially alter the overall conclusions of the meta-analysis regarding CQ 4 (eAppendix and eFigure 13-16 in Supplement 1).

## Discussion

In this study, we found that the sensitivity and specificity of the LLM-assisted citation screening were 0.25 to 1.00 and 0.98 to 0.99, respectively, with studies included by conventional citation screening during the full-text screening session as the reference standard. Moreover, the post hoc analysis using a modified command prompt exhibited higher sensitivity (0.75-1.00) while maintaining the specificity (0.96-0.99). Furthermore, the processing time of the LLM-assisted citation screening method was significantly shorter than that of the conventional method. Few studies have investigated the efficiency and workload reduction of LLM-assisted citation screening in the systematic review process for the development of clinical practice guidelines, and the results of this study may lead to the appropriate utilization of the best evidence.

Our findings indicated the potential of LLM-assisted citation screening, which has substantial advantages over previously reported semiautomated screening tools. First, the LLM-assisted citation screening may lead to improved efficiency and workload reduction during the screening process because although semiautomated citation screening tools using machine learning showed enhanced efficiency and workload reduction, their application requires training data for the citation screening process, inputting predefined key articles, and some processes of human reviewers.^4,9,26^ In contrast, LLM-assisted citation screening does not require any further training data or efforts of human reviewers in the screening process. Our study further found that LLM-assisted citation screening helped save time in the systematic review process, with a more than 10-fold reduction in the time required to complete the process. Although this finding is consistent with other reports showing the advantage of citation screening using semiautomated screening software,^9,10,26^ eliminating the necessity of inputting key studies would save additional time using LLM-assisted citation screening.

Second, LLM-assisted citation may have a higher accuracy than the semiautomated tool. Previous studies on semiautomated citation screening tools reported sensitivity ranging from 0.75 to 0.90,^9,26,27^ which is comparable with the accuracy of our study; however, our previous research on this tool^10^ showed a variable sensitivity of 0.24 to 0.80 for the same dataset used in the present study. Moreover, we found a higher sensitivity of 0.53 to 0.95 with lower variability in the secondary analysis, suggesting the potential advantage of LLM-assisted citation screening for discriminating the relevant literature. Although we found high specificity in primary and secondary analyses, caution is warranted regarding the potential overestimation of the model’s performance owing to the high proportion of true negatives.

Third, LLM-assisted citation screening has other potential advantages, including higher generalizability across various topics and formats, a user-friendly interface to simplify user interaction, continuous development of the model to improve the accuracy over time for each task performed, and functional extensibility to expand its applicability. These advantages support the use of LLMs for citation screening by reducing the workload and maintaining sufficient accuracy, leading to a transformation of the systematic review process.

In our post hoc analysis, the modified prompt improved sensitivity with slightly decreased specificity, suggesting that prompt content may substantially affect the quality of systematic reviews using the LLM. Recent research on prompt engineering has revealed how prompt design influences output, highlighting tactics for enhancing efficiency.^19,20^ In the initial prompt, we described prompt sets according to the list of PICO of the selected clinical questions. Subsequent analysis based on the predetermined study protocol revealed a cautiously low sensitivity for CQ 2 (sensitivity: 0.25; specificity: 0.99). After reviewing the LLM responses to the inadvertently excluded studies (eTable 4 in Supplement 1), we found that the LLM strictly applied the criteria according to the prompt. In the title and abstract screening session, human reviewers tended to be more conservative in their selection of literature to ensure that relevant literature was not excluded. Considering this nature of the title and abstract screening session, such subtle nuances in the prompt commands may have been necessary. Accordingly, we modified the prompt command to loosen the criteria and maximize sensitivity. Upon evaluating the post hoc analysis results, the LLM-assisted citation screening improved in accuracy. Through this modification process, we discovered an optimal description of the citation screening command prompts. While false positives may be somewhat tolerable under certain circumstances, false negatives are more critical, as they signify missed opportunities to include relevant studies, potentially undermining the thoroughness of the systematic review. Consequently, it is imperative to recalibrate the threshold settings to prioritize sensitivity, thereby minimizing the occurrence of false negatives.

To enhance the accuracy of LLM-assisted citation screening, we implemented a majority-vote strategy and a chain-of-thought strategy.^24,25^ The LLM can generate different recommendations across multiple runs, leading to performance uncertainty owing to the probabilistic responses of the LLMs. To ensure the impact of uncertain responses from the LLM on the citation screening performance, we examined the outcomes of the majority-vote strategy. The majority-vote strategy enhanced the sensitivity of the screening sessions using the original and modified prompts, with a slight decrease in specificity. This suggests that this strategy may be promising for improving the accuracy and reliability of citation screening. In addition, the chain-of-thought strategies have been recognized as a prompt engineering technique eliciting accurate responses from LLMs.^25^ However, our post hoc analyses did not demonstrate the effectiveness of this strategy in enhancing precision. Although our investigation was limited to the chain-of-thought strategy’s effects, future research should elucidate the influence of additional prompt engineering techniques, such as the regeneration of superior prompts by LLMs and the implementation of a self-correction strategy, on the performance of LLM-assisted citation screening.

### Limitations

This study has several limitations. First, because our study focused exclusively on a single medical setting and a literature review for clinical guideline development, the applicability of our findings to other fields is uncertain. Future studies should test the LLM-assisted model across various opportunities for systematic review to validate its utility and performance for a wider range of tasks. Second, the quality of the LLM outputs depends on regular model updates, which may vary in frequency and impact, thereby affecting the standardization of review quality over time. Third, the reference standard used in this study was selected by a limited number of members of the J-SSCG 2024 working group, who are experts in the field; however, we cannot rule out the possibility that essential literature was not selected, which may have led to misclassification of the reference standard. Fourth, although the LLM could not access the results of conventional screening, the authors in this study were not masked to the standard reference. Therefore, we registered the study protocol before the analysis to ensure transparency of the performance evaluation. Furthermore, we believe that integrated sensitivity estimates based on primary and secondary analyses are insufficient to support the implementation of this approach in practical settings because this study remains in the proof-of-concept stage. However, we believe that this study provides reasonable evidence justifying further research and validation for practical deployment. Despite these limitations, the integration of advanced artificial intelligence, such as an LLM, into systematic reviews holds great promise, heralding a future with enhanced speed and breadth of knowledge synthesis.

## Conclusions

This prospective diagnostic study found that LLM-assisted citation screening achieved reasonably high specificity, acceptable sensitivity, and reduced processing time. The use of this innovative approach should be further validated to enhance the efficiency and accessibility of systematic review procedures.

## Notes

Supplement 1.
eAppendix.
**eTable 1.** List of the Patient/Population/Problem, Intervention, and Comparison of the Selected Clinical Questions **eTable 2.** Statistics on the Accuracy of Large Language Model–Assisted Citation Screening **eTable 3.** Comparison of Citation Screening Time for 100 Studies per Person Between the Large Language Model–Assisted and Conventional Methods **eTable 4.** List of Unidentified Studies Using the Large Language Model–Assisted Citation Screening for Qualitative Analysis **eTable 5.** Post Hoc Analysis for Evaluating the Accuracy of Large Language Model–Assisted Citation Screening **eFigure 1.** Command Prompt for the LLM Citation Screening Task **eFigure 2.** Comparison of Citation Screening Time for 100 Studies Between the Large Language Model–Assisted and Conventional Methods **eFigure 3.** Modified Command Prompt for the LLM Citation Screening Task in the Post Hoc Analysis **eFigure 4.** Post Hoc Analysis for the Secondary Analysis Using the Modified Prompt **eFigure 5.** Post Hoc Analysis for the Primary Analysis Using the Original Prompt and a Majority-Vote Strategy **eFigure 6.** Post Hoc Analysis for the Secondary Analysis Using the Original Prompt and a Majority-Vote Strategy **eFigure 7.** Post Hoc Analysis for the Secondary Analysis Using the Modified Prompt and a Majority Vote-Strategy **eFigure 8.** Modified Command Prompt Integrating the Chain-of-Thought Strategy for the LLM Citation Screening Task in the Post Hoc Analysis **eFigure 9.** Post Hoc Analysis for the Primary Analysis Using the Original Prompt and the Chain-of-Thought Strategy **eFigure 10.** Post Hoc Analysis for the Secondary Analysis Using the Original Prompt and the Chain-Of-Thought Strategy **eFigure 11.** Post Hoc Analysis for the Primary Analysis Using the Modified Prompt and the Chain-of-Thought Strategy **eFigure 12.** Post Hoc Analysis for the Secondary Analysis Using the Modified Prompt and the Chain-of-Thought Strategy **eFigure 13.** Forest Plots of Pairwise Meta-Analyses for Short-Term Mortality **eFigure 14.** Forest Plots of Pairwise Meta-Analyses for ICU Mortality **eFigure 15.** Forest Plots of Pairwise Meta-Analyses for ICU Length Of Stay **eFigure 16.** Forest Plots of Pairwise Meta-Analyses for Ventilator-Free Days
eReferences.

Supplement 2.
Data Sharing Statement
