# Supplementary material for: Performance of a Large Language Model in Screening Citations
Source: JAMA Netw Open. 2024 Jul 8;7(7):e2420496. doi: 10.1001/jamanetworkopen.2024.20496 (PMC11231796; doi:10.1001/jamanetworkopen.2024.20496)
Supplement: Supplement 1. — eAppendix. eTable 1. List of the Patient/Population/Problem, Intervention, and Comparison of the Selected Clinical Questions eTable 2. Statistics on the Accuracy of Large Language Model–Assisted Citation Screening eTable 3. Comparison of Citation Screening Time for 100 Studies per Person Between the Large Language Model–Assisted and Conventional Methods eTable 4. List of Unidentified Studies Using the Large Language Model–Assisted Citation Screening for Qualitative Analysis eTable 5. Post Hoc Analysis for Evaluating the Accuracy of Large Language Model–Assisted Citation Screening eFigure 1. Command Prompt for the LLM Citation Screening Task eFigure 2. Comparison of Citation Screening Time for 100 Studies Between the Large Language Model–Assisted and Conventional Methods eFigure 3. Modified Command Prompt for the LLM Citation Screening Task in the Post Hoc Analysis eFigure 4. Post Hoc Analysis for the Secondary Analysis Using the Modified Prompt eFigure 5. Post Hoc Analysis for the Primary Analysis Using the Original Prompt and a Majority-Vote Strategy eFigure 6. Post Hoc Analysis for the Secondary Analysis Using the Original Prompt and a Majority-Vote Strategy eFigure 7. Post Hoc Analysis for the Secondary Analysis Using the Modified Prompt and a Majority Vote-Strategy eFigure 8. Modified Command Prompt Integrating the Chain-of-Thought Strategy for the LLM Citation Screening Task in the Post Hoc Analysis eFigure 9. Post Hoc Analysis for the Primary Analysis Using the Original Prompt and the Chain-of-Thought Strategy eFigure 10. Post Hoc Analysis for the Secondary Analysis Using the Original Prompt and the Chain-Of-Thought Strategy eFigure 11. Post Hoc Analysis for the Primary Analysis Using the Modified Prompt and the Chain-of-Thought Strategy eFigure 12. Post Hoc Analysis for the Secondary Analysis Using the Modified Prompt and the Chain-of-Thought Strategy eFigure 13. Forest Plots of Pairwise Meta-Analyses for Short-Term Mortality eFigure 14. Forest Plots of Pairw [file jamanetwopen-e2420496-s001.pdf]

Supplemental Online Content

Oami T, Okada Y, Nakada T. Performance of a large language model in screening citations. *JAMA Netw Open.* 2024;7(7):e2420496. doi:10.1001/jamanetworkopen.2024.20496

eAppendix.

eTable 1. List of the Patient/Population/Problem, Intervention, and Comparison of the Selected Clinical Questions

eTable 2. Statistics on the Accuracy of Large Language Model–Assisted Citation Screening

eTable 3. Comparison of Citation Screening Time for 100 Studies per Person Between the Large Language Model–Assisted and Conventional Methods

eTable 4. List of Unidentified Studies Using the Large Language Model–Assisted Citation Screening for Qualitative Analysis

eTable 5. Post Hoc Analysis for Evaluating the Accuracy of Large Language Model–Assisted Citation Screening

eFigure 1. Command Prompt for the LLM Citation Screening Task

eFigure 2. Comparison of Citation Screening Time for 100 Studies Between the Large Language Model–Assisted and Conventional Methods

eFigure 3. Modified Command Prompt for the LLM Citation Screening Task in the Post Hoc Analysis

eFigure 4. Post Hoc Analysis for the Secondary Analysis Using the Modified Prompt

eFigure 5. Post Hoc Analysis for the Primary Analysis Using the Original Prompt and a Majority-Vote Strategy

eFigure 6. Post Hoc Analysis for the Secondary Analysis Using the Original Prompt and a Majority-Vote Strategy

eFigure 7. Post Hoc Analysis for the Secondary Analysis Using the Modified Prompt and a Majority Vote-Strategy

eFigure 8. Modified Command Prompt Integrating the Chain-of-Thought Strategy for the LLM Citation Screening Task in the Post Hoc Analysis

eFigure 9. Post Hoc Analysis for the Primary Analysis Using the Original Prompt and the Chain-of-Thought Strategy

eFigure 10. Post Hoc Analysis for the Secondary Analysis Using the Original Prompt and the Chain-Of-Thought Strategy

eFigure 11. Post Hoc Analysis for the Primary Analysis Using the Modified Prompt and the Chain-of-Thought Strategy

© 2024 Oami T et al. *JAMA Network Open.*

eFigure 12. Post Hoc Analysis for the Secondary Analysis Using the Modified Prompt and the Chain-of-Thought Strategy

eFigure 13. Forest Plots of Pairwise Meta-Analyses for Short-Term Mortality

eFigure 14. Forest Plots of Pairwise Meta-Analyses for ICU Mortality

eFigure 15. Forest Plots of Pairwise Meta-Analyses for ICU Length Of Stay

eFigure 16. Forest Plots of Pairwise Meta-Analyses for Ventilator-Free Days

eReferences.

This supplemental material has been provided by the authors to give readers additional information about their work.

© 2024 Oami T et al. *JAMA Network Open.*

eAppendix

Table of Contents

1. Clinical questions in the Japanese Clinical Practice Guidelines for Management of Sepsis and Septic Shock

2. Conventional citation screening

3. Statistics on accuracy of the LLM-assisted citation screening in the secondary analysis

4. Overall citation screening time for 100 studies between the LLM-assisted and conventional methods

5. Modified command based on the false negative studies

6. Statistics on accuracy of the LLM-assisted citation screening in the *post hoc* analysis

7. Impact of LLM-based methods on the outcomes of the meta-analyses

© 2024 Oami T et al. *JAMA Network Open.*

eAppendix

1. Clinical questions in the Japanese Clinical Practice Guidelines for Management of Sepsis and Septic Shock

Briefly, members of the J-SSCG 2024 working group conducted extensive literature searches for these clinical questions (CQs) using CENTRAL, PubMed, and Ichushi-Web (the Japanese biomedical literature database), with a comprehensive search strategy that included all key studies. The literature was restricted to articles written in Japanese and English. Subsequently, all titles and abstracts were downloaded, organized, and deduplicated using EndNote (Clarivate Analytics, Philadelphia, PA, USA) for citation management for J-SSCG 2024.[1]

2. Conventional citation screening

EndNote-processed files were imported into Rayyan,[2] a web application designed to facilitate systematic reviews. Subsequently, two independent reviewers screened titles and abstracts, resolving any discrepancies either by consensus or by the judgment of a third reviewer. The literature selected through these conventional citation screening methods was used as a reference standard. The detailed of the conventional citation screening are described in the Supplementary File. Notably, conventional screening results are independent of the large language model (LLM)-assisted citation screening process and are concealed from the LLM in the current analysis to preserve the integrity of the evaluation process. In addition, the authors of this study were not involved in the conventional literature search of the selected CQs. We used previously published data on the duration of the screening session recorded using Rayyan's built-in time-tracking feature to obtain the time required to conduct the conventional screening method.[2,3]

3. Statistics on accuracy of the LLM-assisted citation screening in the secondary analysis

LLM-assisted citation screening selected 38 publications in CQ1 (0.67%), 9 in CQ2 (0.26%), 5 in CQ3 (0.48%), 37 in CQ4 (0.86%), and 27 in CQ5 (1.20%) in the title/abstract screening session. In the secondary analysis, the sensitivity and specificity with 95% CI obtained from the LLM-assisted screening were 0.34 [0.26–0.43] and 1.00 [1.00–1.00] for CQ1, 0.53 [0.30–0.74] and 1.00 [1.00–1.00] for CQ2, 0.36 [0.16–0.62] and 1.00 [0.99–1.00] for CQ3, 0.53 [0.41–0.64] and 0.99 [0.98–0.99] for CQ4, and 0.69 [0.53–0.82] and 0.99 [0.99–0.99] for CQ5, respectively (Figure 3).

4. Overall citation screening time for 100 studies between the LLM-assisted and conventional methods

Using the conventional citation screening approach, the median [minimum to maximum] time (min) lapsed to screen 100 studies for each CQ during the title/abstract screening was 14.7 [13.1, 18.7] min for CQ1, 11.9 [10.0, 31.1] min for CQ2, 16.3 [7.9, 31.2] min for CQ3, 15.8 [13.1, 27.7] min for CQ4, and 11.7 [10.5, 17.1] min for CQ5. The processing time per study for each CQ in the LLM-assisted citation screening was 1.3 min for CQ1, 1.3 min for CQ2, 1.3 min for CQ3, 1.3 min for CQ4, and 1.3 min for CQ5 (eTable 3 in the Supplement).

5. Modified command

In the primary analyses, we observed three false-negative publications in CQ2, two in CQ3, and three in CQ4 (eTable 2 in the Supplement). When considering why the LLM opted for exclusion, we found that the LLM strictly followed the inclusion criteria according to the CQ framework (eTable 4 in the Supplement). Therefore, we modified the command prompt to make the inclusion more flexible to increase the sensitivity of LLM-assisted citation screening (eFigure 3 in the Supplement).

6. Statistics on accuracy of the LLM-assisted citation screening in the post hoc analysis

© 2024 Oami T et al. *JAMA Network Open.*

The sensitivity and specificity with 95% CI were 1.00 [0.50–1.00] and 0.99 [0.98–0.99] for CQ1, 1.00 [0.33–0.99] and 0.98 [0.98–0.99] for CQ2, 0.75 [0.24–0.97] and 0.99 [0.98–1.00] for CQ3, 0.88 [0.63–0.97] and 0.96 [0.96–0.97] for CQ4, and 1.00 [0.50–1.00] and 0.97 [0.96–0.98] for CQ5, respectively, in the primary analysis (Fig. 4).

The sensitivity and specificity values with 95% CI were 0.53 [0.44–0.62] and 1.00 [0.99–1.00] for CQ1, 0.82 [0.57–0.94] and 0.99 [0.98–0.99] for CQ2, 0.64 [0.38–0.84] and 1.00 [0.99–1.00] for CQ3, 0.63 [0.51–0.73] and 0.97 [0.97–0.98] for CQ4, and 0.95 [0.82–0.99] and 0.98 [0.98–0.99)] for CQ5, respectively, in the secondary analysis (eFig. 4 in the Supplement).

7. Impact of LLM-based methods on the outcomes of the meta-analyses

On April 17, 2024, only the results of the meta-analysis for CQ4 were publicly available.[4] Thus, we conducted a meta-analysis for CQ4, excluding studies identified as false negatives in LLM-assisted screening, and compared these findings with those obtained using conventional methods.

© 2024 Oami T et al. *JAMA Network Open.*

eTable 1. List of the Patient/Population/Problem, Intervention, and Comparison of the Selected Clinical Questions

	Patient, population, problem	Intervention	Comparison
CQ1	Adult patients (18 years old or older) diagnosed with or suspected of having infection, bacteremia, or sepsis	Balanced crystalloid administration	0.9% sodium chloride administration
CQ2	Adult patients (18 years old or older) with sepsis or suspected as sepsis, infection, bacteremia or patients admitted to ICU	Targeting a higher mean arterial pressure	Targeting a lower mean arterial pressure
CQ3	Adult patients (18 years old or older) with sepsis presenting with severe metabolic acidosis or patients admitted to ICU	Sodium bicarbonate administration	No sodium bicarbonate administration
CQ4	Adult patients (18 years old or older) with sepsis or septic shock	Usual care with at least one of the following tissue perfusion parameters: lactate/lactate clearance, capillary refill time, $ScvO_2$/SvO_2, and P(v-a) CO2/C (a-v) O_2.	Usual care with different parameters mentioned in the interventional group or standard care without the utilization of any specific tissue perfusion parameters
CQ5	Adult patients (18 years old or older) with sepsis, sepsis-induced hypotension, or septic shock	Restrictive fluid management aiming to reduce the amount of fluid therapy for up to 24 h	Conventional fluid management or non-restrictive fluid management defined by authors

CQ: clinical question; ICU: intensive care unit

© 2024 Oami T et al. *JAMA Network Open.*

eTable 2. Statistics on the Accuracy of Large Language Model–Assisted Citation Screening

	TN	FP	FN	TP
Primary analysis[a]				
CQ1	5,577	49	0	8
CQ2	3,398	16	3	1
CQ3	1,028	6	2	2
CQ4	4,283	26	3	14
CQ5	2,209	36	0	8
Secondary analysis[b]				
CQ1	5,503	19	74	38
CQ2	3,393	8	8	9
CQ3	1,027	3	9	5
CQ4	4,197	59	33	37
CQ5	2,197	17	12	27

CQ, clinical question; FN, false negative; FP, false positive; TN, true negative; TP, true positive

[a] The list of included studies for qualitative analysis using the conventional method was set as the standard reference.
[b] The list of included studies after the title/abstract screening using the conventional method was set as a standard reference.

© 2024 Oami T et al. *JAMA Network Open.*

eTable 3. Comparison of Citation Screening Time for 100 Studies per Person Between the Large Language Model–Assisted and Conventional Methods

	LLM-assisted citation screening	Conventional citation screening		
	Time (min)	Median (min)	Min (min)	Max (min)
CQ1	1.3	14.7	13.1	18.7
CQ2	1.3	11.9	10.0	31.1
CQ3	1.3	16.3	7.9	31.2
CQ4	1.3	15.8	13.1	27.7
CQ5	1.3	11.7	10.5	17.1

LLM: large language model, CQ: clinical question

© 2024 Oami T et al. *JAMA Network Open.*

eTable 4. List of Unidentified Studies Using the Large Language Model–Assisted Citation Screening for Qualitative Analysis

No.	CQ	Year	Journal	Title	The reason for exclusion
1[a]	CQ2	2020	JAMA	Effect of Reduced Exposure to Vasopressors on 90-Day Mortality in Older Critically Ill Patients with Vasodilatory Hypotension: A Randomized Clinical Trial	The study focused on older critically ill patients (aged 65 years or older). Our systematic review and meta-analysis required the inclusion of adult patients aged 18 or older without an upper age limit specified. This study did not meet the criteria as it exclusively considered an older patient cohort. Moreover, it investigated permissive hypotension rather than targeting a higher mean arterial pressure compared with a lower one, which did not align with the intervention criteria for our review.
2[a]	CQ2	2016	Intensive Care Med	Higher versus lower blood pressure targets for vasopressor therapy in shock: a multicenter pilot randomized controlled trial	The study population included patients with vasodilatory shock regardless of admission diagnosis, which meant not all patients may have sepsis, suspected sepsis, infection, bacteremia, or were necessarily admitted to the ICU owing to these conditions. The study must specifically include patients that fit the target patient criteria outlined for the systematic review.
3[a]	CQ2	2021	Health Technol Assess	Reduced exposure to vasopressors through permissive hypotension to reduce mortality in critically ill people aged 65 and over: the 65 RCT	The study focused on critically ill patients aged 65 and over. Our inclusion criteria specified adult patients with sepsis or related conditions but did not limit the study population to a specific age group beyond being 18 years or older. The study in question exclusively included an older patient population, thus not representing the broader adult population we were interested in.
4	CQ3	1991	Crit Care Med	Effects of bicarbonate therapy on hemodynamics	The study was designed as a prospective, randomized, blinded, cross over study, which is a type of randomized controlled trial; however, the patient population did not

© 2024 Oami T et al. *JAMA Network Open.*

				and tissue oxygenation in patients with lactic acidosis: a prospective, controlled clinical study	specifically mention patients with sepsis or ICU admission. Only patients with metabolic acidosis and increased arterial plasma lactate were included. Since the patient eligibility criteria were not fully met, this study was excluded.
5[a]	CQ3	1990	Ann Intern Med	Bicarbonate does not improve hemodynamics in critically ill patients who have lactic acidosis. A prospective, controlled clinical study	The study was designed as a crossover study, which is not the same as a simple randomized controlled trial (RCT). This design introduced an additional layer of complexity where each patient receives both the intervention and the control, which was not consistent with the simple parallel-group RCT structure required as per the eligibility criteria.
6[a]	CQ4	2007	Nan Fang Yi Ke Da Xue Xue Bao	Early goal-directed therapy lowers the incidence, severity and mortality of multiple organ dysfunction syndrome	The primary outcome of the study was the incidence, severity, and mortality of multiple organ dysfunction syndrome (MODS), not sepsis or septic shock specifically. Although MODS could be a consequence of severe sepsis and patients with severe sepsis might have been included in the study, the criteria for inclusion required the study to focus on adult patients with sepsis or septic shock. Additionally, it was not clear if the intervention included tissue perfusion parameters mentioned in the criteria (lactate clearance, capillary refill time, ScvO2/SvO2, and P(v-a) CO2/C (a-v) O2) as part of the EGDT protocol. The abstract mentioned blood lactate concentration but did not specify whether the EGDT protocol adhered to the specific perfusion parameters criteria required for inclusion in the review.
7	CQ4	1995	New England	A TRIAL OF GOAL-ORIENTED HEMODYNAMIC	The study included critically ill adult patients and investigated the effects of hemodynamic therapy on outcomes but did not specifically state that the patient population had sepsis or septic shock. The intervention also did not mention the tissue

© 2024 Oami T et al. *JAMA Network Open.*

			Journal of Medicine	THERAPY IN CRITICALLY ILL PATIENTS	perfusion parameters outlined in the inclusion criteria (lactate/lactate clearance, capillary refill time, ScvO2/SvO2, P(v-a) CO2/C (a-v) O2). Instead, the study focused on achieving supranormal levels of the cardiac index or normal levels of mixed venous oxygen saturation, which were not among the specified parameters. Therefore, the study did not meet the eligibility criteria for the systematic review and meta-analysis.
8	CQ4	2012	Zhongguo wei zhong bing ji jiu yi xue [Chinese critical care medicine]	The effect of early goal lactate clearance rate on the outcome of septic shock patients with severe pneumonia	The study was described as a randomized perspective study, indicating a prospective study design. However, it was not explicitly stated as a randomized controlled trial (RCT), which is a specific requirement for inclusion in the review. The absence of clear confirmation that the study was a randomized controlled trial necessitated its exclusion based on the predefined eligibility criteria.

LLM: large language model, CQ: clinical question

[a] Included studies using a modified command prompt for the *post hoc* study

© 2024 Oami T et al. *JAMA Network Open.*

eTable 5. Post Hoc Analysis for Evaluating the Accuracy of Large Language Model–Assisted Citation Screening[a]

A. Modified prompt based on the false negative studies

	TN	FP	FN	TP
Primary analysis[a]				
CQ1	5,549	77	0	8
CQ2	3,355	59	0	4
CQ3	1,026	8	1	3
CQ4	4,160	149	2	15
CQ5	2,178	67	0	8
Secondary analysis[b]				
CQ1	5,477	45	72	40
CQ2	3,352	49	3	14
CQ3	1,022	2	5	9
CQ4	4,136	120	26	44
CQ5	2,176	38	2	37

B. Majority vote strategy using the original prompt

	TN	FP	FN	TP
Primary analysis[a]				
CQ1	5,569	57	0	8
CQ2	3,391	23	3	1
CQ3	1,028	6	2	2
CQ4	4,186	123	3	14
CQ5	2,193	52	0	8
Secondary analysis[b]				
CQ1	5,495	27	74	38
CQ2	3,387	14	7	10
CQ3	1,021	3	9	5
CQ4	4,162	94	27	43
CQ5	2,187	27	6	33

C. Majority vote strategy using the modified prompt

	TN	FP	FN	TP
Primary analysis[a]				
CQ1	5,544	82	0	8
CQ2	3,320	94	0	4
CQ3	1,022	12	1	3
CQ4	4,076	233	1	16
CQ5	2,176	69	0	8
Secondary analysis[b]				
CQ1	5,488	34	56	56
CQ2	3,318	83	2	15
CQ3	1,019	5	4	10
CQ4	4,057	199	20	50
CQ5	2,175	39	1	38

© 2024 Oami T et al. *JAMA Network Open.*

D. Chain of thought strategy using the original prompt

	TN	FP	FN	TP
Primary analysis[a]				
CQ1	5,578	48	1	7
CQ2	3,397	17	3	1
CQ3	1,028	6	2	2
CQ4	4,219	90	4	13
CQ5	2,201	44	0	8
Secondary analysis[b]				
CQ1	5,499	23	80	32
CQ2	3,391	10	9	8
CQ3	1,021	3	9	5
CQ4	4,192	64	31	39
CQ5	2,191	23	10	29

E. Chain of thought strategy using the modified prompt

	TN	FP	FN	TP
Primary analysis[a]				
CQ1	5,540	86	0	8
CQ2	3,346	68	0	4
CQ3	1,022	12	2	2
CQ4	4,116	193	1	16
CQ5	2,175	70	0	8
Secondary analysis[b]				
CQ1	5,476	46	64	48
CQ2	3,343	58	3	14
CQ3	1,019	5	5	9
CQ4	4,095	161	22	48
CQ5	2,173	41	2	37

CQ, clinical question; FN, false negative; FP, false positive; TN, true negative; TP, true positive

[a] The list of included studies for qualitative analysis using the conventional method was set as the standard reference.
[b] The list of included studies after the title/abstract screening using the conventional method was set as the standard reference.

© 2024 Oami T et al. *JAMA Network Open.*

eFigure 1. Command Prompt for the LLM Citation Screening Task

You

You are conducting a systematic review and meta-analysis, focusing on a specific area of medical research. Your task is to evaluate research studies and determine whether they should be included in your review. To do this, each study must meet the following criteria:

Target Patients: Adult patients (18 years old or older) diagnosed with or suspected of having infection, bacteremia, or sepsis.
Intervention: The study investigates the effects of balanced crystalloid administration.
Comparison: The study compares the above intervention with 0.9% sodium chloride administration.
Study Design: The study must be a randomized controlled trial.
Additionally, any study protocol that meets these criteria should also be included.

However, you should exclude studies in the following cases:

The study does not meet all of the above eligibility criteria.
The study's design is not a randomized controlled trial. Examples of unacceptable designs include case reports, observational studies, systematic reviews, review articles, animal experiments, letters to editors, and textbooks.
After reading the title and abstract of a study, you will decide whether to include or exclude it based on these criteria. Please answer with include or exclude only.

Title: ---------------------

Abstract

ChatGPT

Include

The content of the command prompt for the GPT-4 citation screening task included the patient/population/problem, intervention, comparison, and study design of clinical questions (CQ). This figure shows an example for CQ1.

© 2024 Oami T et al. *JAMA Network Open.*

eFigure 2. Comparison of Citation Screening Time for 100 Studies Between the Large Language Model–Assisted and Conventional Methods

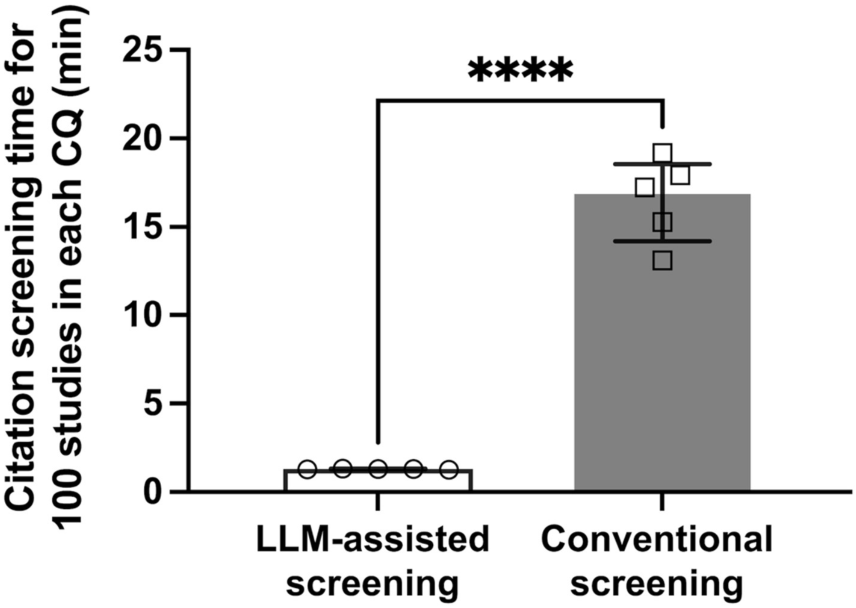

The difference in processing time was −15.25 min (95% confidence interval [−17.70 to −12.79], $p < 0.001$). An unpaired *t*-test was used for the analysis.

© 2024 Oami T et al. *JAMA Network Open.*

eFigure 3. Modified Command Prompt for the LLM Citation Screening Task in the Post Hoc Analysis

You

You are conducting a systematic review and meta-analysis, focusing on a specific area of medical research. Your task is to evaluate research studies and determine whether they should be included in your review. To do this, each study must meet the following criteria:

Target Patients: The study includes adult patients diagnosed with or suspected of having infection, bacteremia, or sepsis. If there is a possibility that the study population includes patients with sepsis, the study should be included.
Intervention: The study investigates the effects of balanced crystalloid administration.
Comparison: The study compares the above intervention with 0.9% sodium chloride administration.
Study Design: The study must be a randomized controlled trial.
Additionally, any study protocol that meets these criteria should also be included.

However, you should exclude studies in the following cases:

The study does not meet all of the above eligibility criteria.
The study's design is not a randomized controlled trial. Examples of unacceptable designs include case reports, observational studies, systematic reviews, review articles, animal experiments, letters to editors, and textbooks.
After reading the title and abstract of a study, you will decide whether to include or exclude it based on these criteria. If there is uncertainty in the decision due to a lack of adequate information as you evaluate each domain, you will answer include to minimize the possibility of inadvertently excluding potentially relevant literature. Please answer with include or exclude only.

Title: ---------------------

Abstract

ChatGPT

Include

The modified command prompt for each clinical question (CQ) includes additional descriptions to the original version of the command prompt, highlighted in red character. This figure shows an example for CQ1.

© 2024 Oami T et al. *JAMA Network Open.*

eFigure 4. Post Hoc Analysis for the Secondary Analysis Using the Modified Prompt

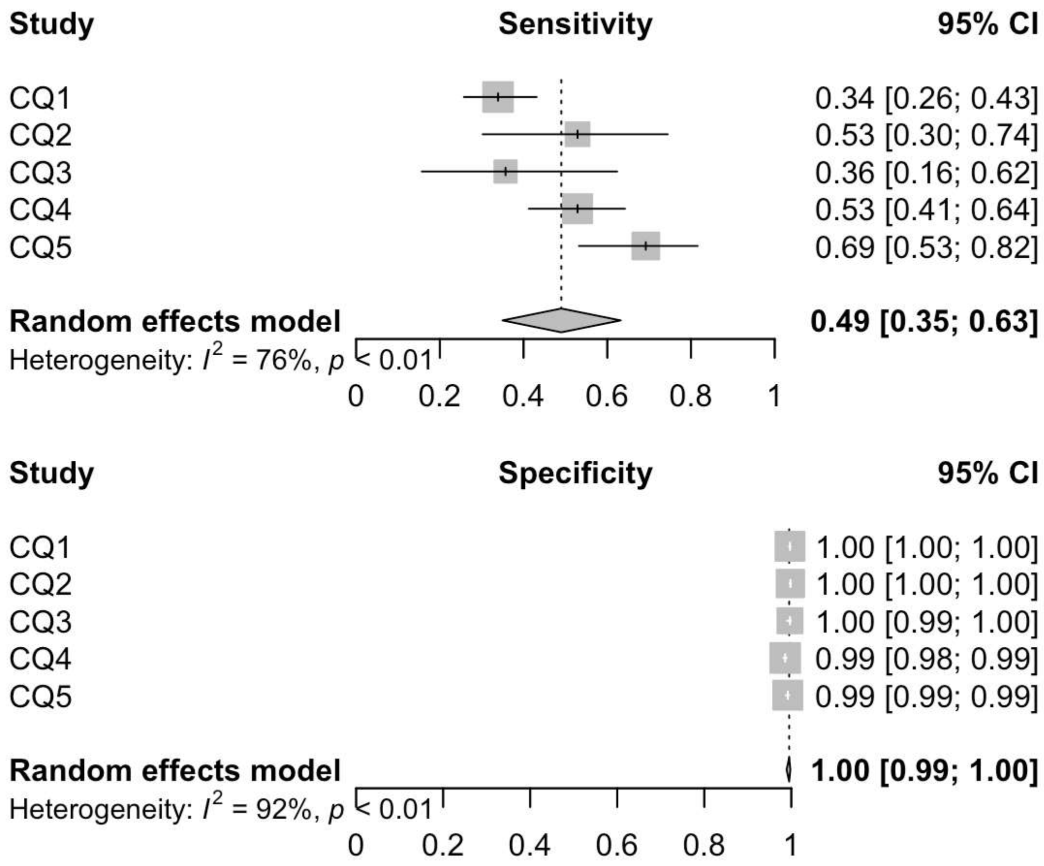

Post hoc secondary analysis adopted the modified prompt based on the false negative studies. The results of the included publications for the full text screening session using the conventional method were used as the standard reference. In the upper panel, the individual sensitivity for each clinical question (CQ) and integrated sensitivities across CQ1-5 are shown, with confidence intervals and inconsistency values (I^2). In the lower panel, the individual specificity for each CQ and integrated specificities across CQ1-5 are shown, with confidence intervals and inconsistency values (I^2).

© 2024 Oami T et al. *JAMA Network Open.*

eFigure 5. Post Hoc Analysis for the Primary Analysis Using the Original Prompt and a Majority-Vote Strategy

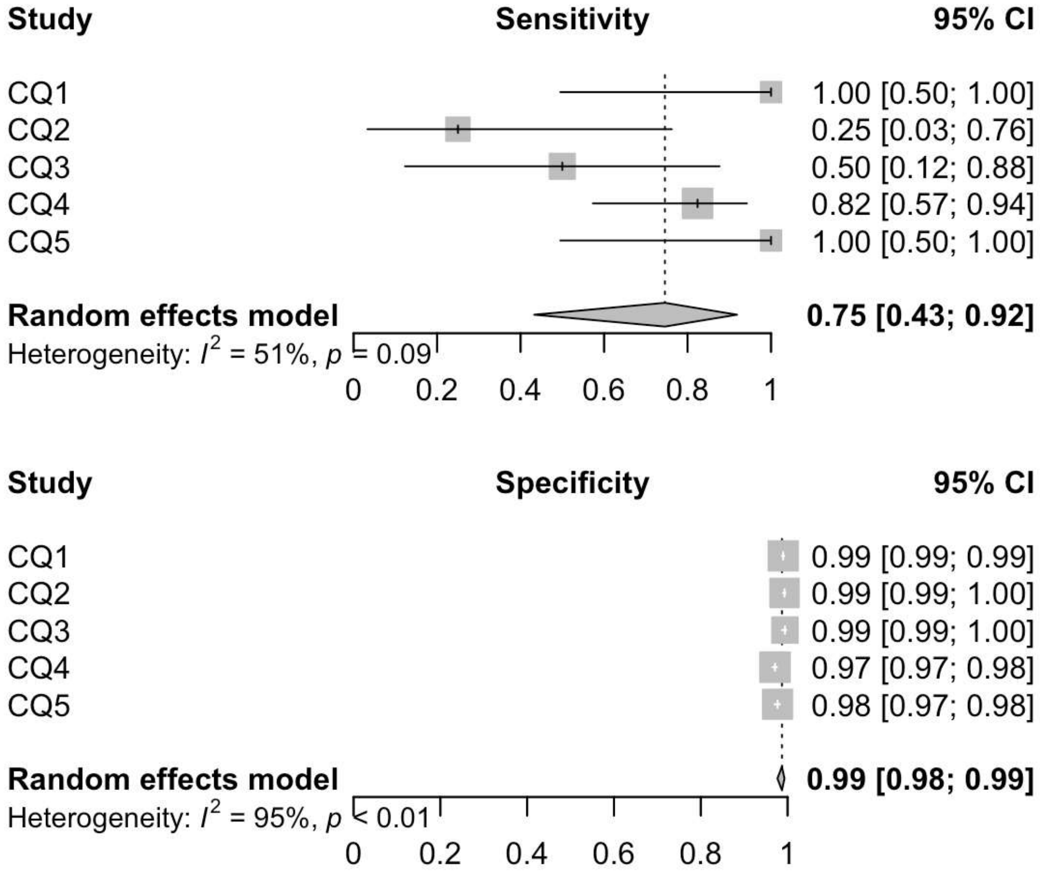

Post hoc primary analysis adopted the original prompt and a majority vote strategy. The results of the included publications for qualitative analysis using the conventional method were used as the standard reference. In the upper panel, the individual sensitivity for each clinical question (CQ) and integrated sensitivities across CQ1-5 are shown, with confidence intervals and inconsistency values (I^2). In the lower panel, the individual specificity for each CQ and integrated specificities across CQ1-5 are shown, with confidence intervals and inconsistency values (I^2).

© 2024 Oami T et al. *JAMA Network Open.*

eFigure 6. Post Hoc Analysis for the Secondary Analysis Using the Original Prompt and a Majority-Vote Strategy

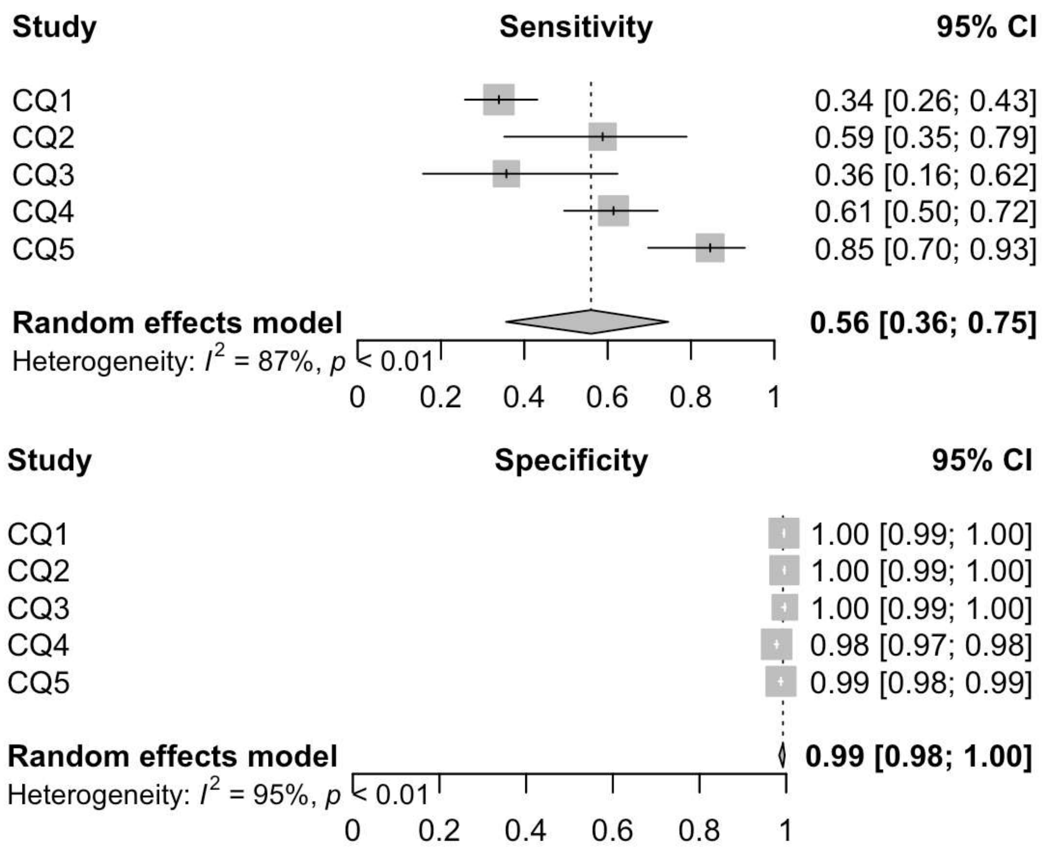

Post hoc secondary analysis adopted the original prompt and a majority vote strategy. The results of the included publications for the full text screening session using the conventional method were used as the standard reference. In the upper panel, the individual sensitivity for each clinical question (CQ) and integrated sensitivities across CQ1-5 are shown, with confidence intervals and inconsistency values (I^2). In the lower panel, the individual specificity for each CQ and integrated specificities across CQ1-5 are shown, with confidence intervals and inconsistency values (I^2).

© 2024 Oami T et al. *JAMA Network Open.*

eFigure 7. Post Hoc Analysis for the Secondary Analysis Using the Modified Prompt and a Majority Vote-Strategy

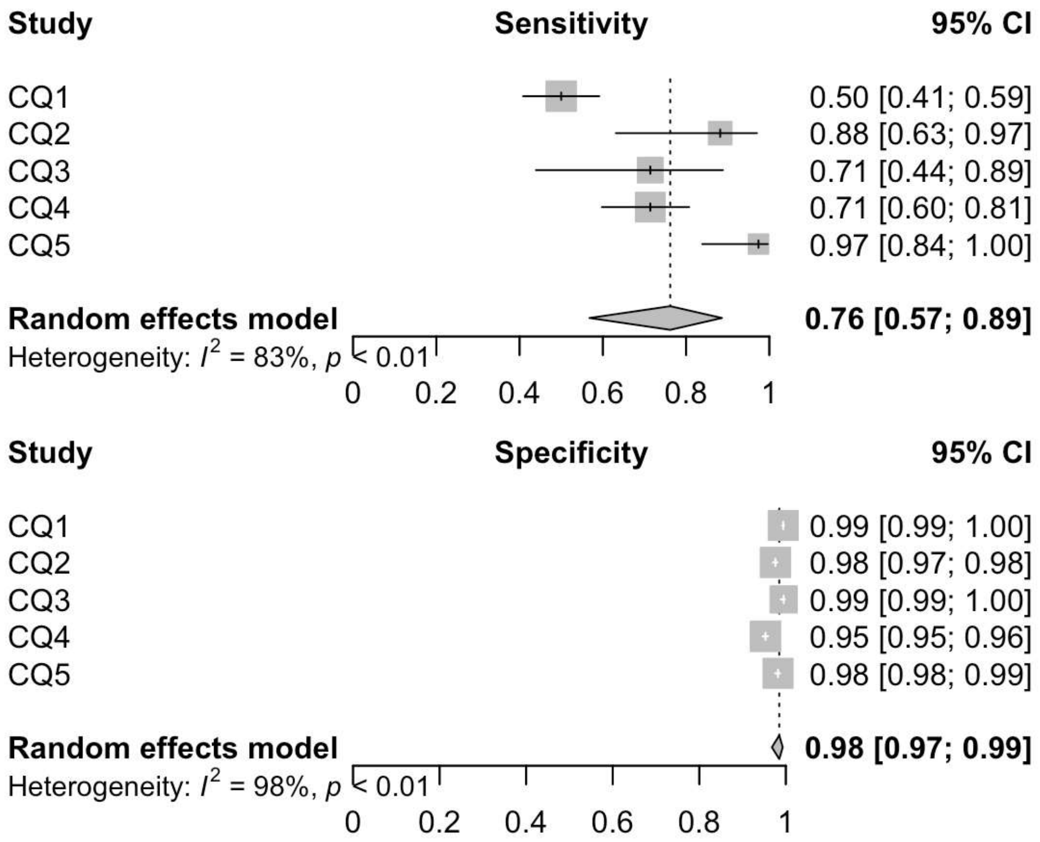

Post hoc secondary analysis adopted the original prompt and a majority vote strategy. The results of the included publications for the full text screening session using the conventional method were used as the standard reference. In the upper panel, the individual sensitivity for each clinical question (CQ) and integrated sensitivities across CQ1-5 are shown, with confidence intervals and inconsistency values (I^2). In the lower panel, the individual specificity for each CQ and integrated specificities across CQ1-5 are shown, with confidence intervals and inconsistency values (I^2).

© 2024 Oami T et al. *JAMA Network Open.*

eFigure 8. Modified Command Prompt Integrating the Chain-of-Thought Strategy for the LLM Citation Screening Task in the Post Hoc Analysis

You

You are conducting a systematic review and meta-analysis, focusing on a specific area of medical research. Your task is to evaluate research studies and determine whether they should be included in your review. To do this, each study must meet the following criteria:

Target Patients: Adult patients (18 years old or older) diagnosed with or suspected of having infection, bacteremia, or sepsis.
Intervention: The study investigates the effects of balanced crystalloid administration.
Comparison: The study compares the above intervention with 0.9% sodium chloride administration.
Study Design: The study must be a randomized controlled trial.
Additionally, any study protocol that meets these criteria should also be included.

However, you should exclude studies in the following cases:

The study does not meet all of the above eligibility criteria.
The study's design is not a randomized controlled trial. Examples of unacceptable designs include case reports, observational studies, systematic reviews, review articles, animal experiments, letters to editors, and textbooks.
After reading the title and abstract of a study, you will decide whether to include or exclude it based on these criteria. Let's think step by step. Please answer with include or exclude only.

Title: ----------------------

Abstract

ChatGPT

Include

© 2024 Oami T et al. *JAMA Network Open.*

eFigure 9. Post Hoc Analysis for the Primary Analysis Using the Original Prompt and the Chain-of-Thought Strategy

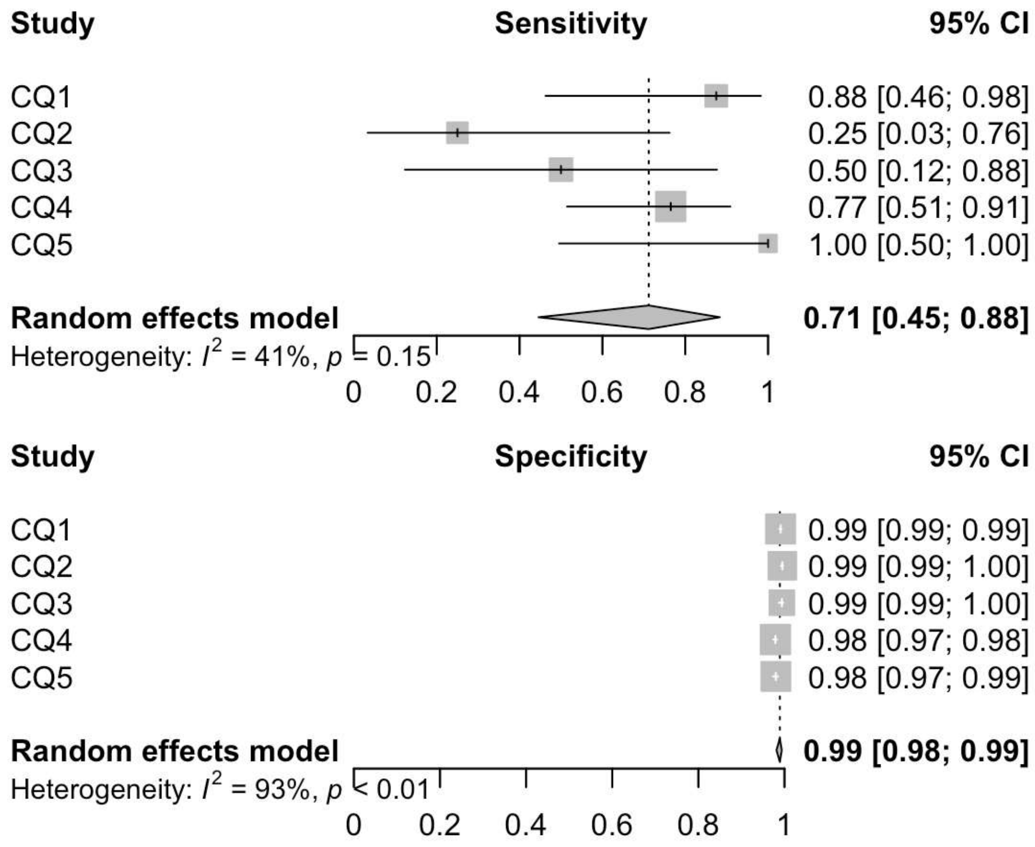

Post hoc secondary analysis adopted the original prompt and the chain of thought strategy. The results of the included publications for the full text screening session using the conventional method were used as the standard reference. In the upper panel, the individual sensitivity for each clinical question (CQ) and integrated sensitivities across CQ1-5 are shown, with confidence intervals and inconsistency values (I^2). In the lower panel, the individual specificity for each CQ and integrated specificities across CQ1-5 are shown, with confidence intervals and inconsistency values (I^2).

© 2024 Oami T et al. *JAMA Network Open.*

eFigure 10. Post Hoc Analysis for the Secondary Analysis Using the Original Prompt and the Chain-Of-Thought Strategy

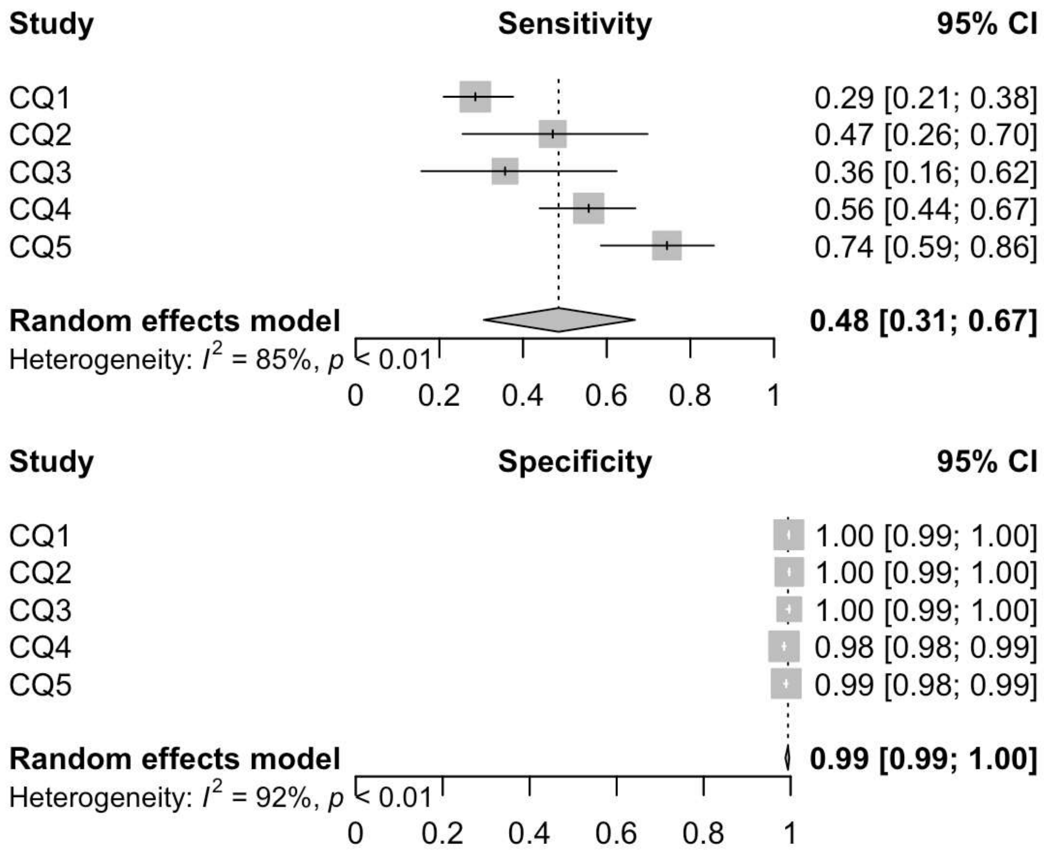

Post hoc primary analysis adopted the original prompt and the chain of thought strategy. The results of the included publications for qualitative analysis using the conventional method were used as the standard reference. In the upper panel, the individual sensitivity for each clinical question (CQ) and integrated sensitivities across CQ1-5 are shown, with confidence intervals and inconsistency values (I^2). In the lower panel, the individual specificity for each CQ and integrated specificities across CQ1-5 are shown, with confidence intervals and inconsistency values (I^2).

© 2024 Oami T et al. *JAMA Network Open.*

eFigure 11. Post Hoc Analysis for the Primary Analysis Using the Modified Prompt and the Chain-of-Thought Strategy

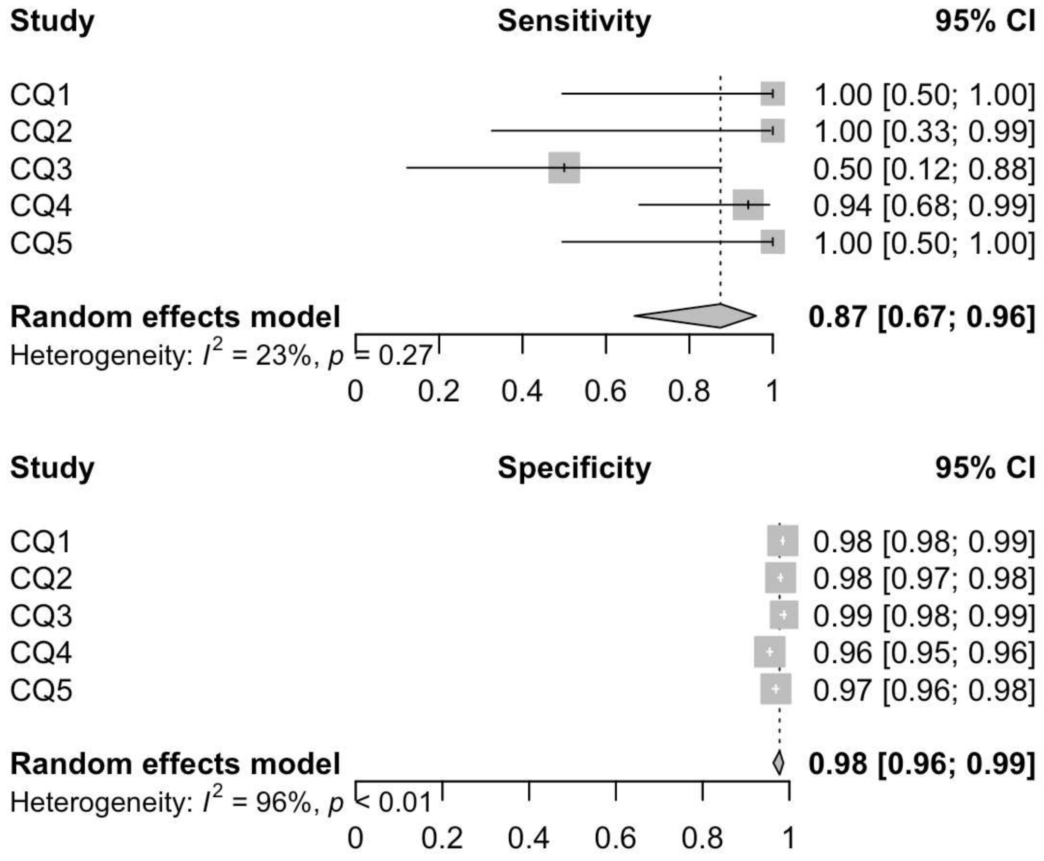

Post hoc primary analysis adopted the modified prompt and the chain of thought strategy. The results of the included publications for qualitative analysis using the conventional method were used as the standard reference. In the upper panel, the individual sensitivity for each clinical question (CQ) and integrated sensitivities across CQ1-5 are shown, with confidence intervals and inconsistency values (I^2). In the lower panel, the individual specificity for each CQ and integrated specificities across CQ1-5 are shown, with confidence intervals and inconsistency values (I^2).

© 2024 Oami T et al. *JAMA Network Open.*

eFigure 12. Post Hoc Analysis for the Secondary Analysis Using the Modified Prompt and the Chain-of-Thought Strategy

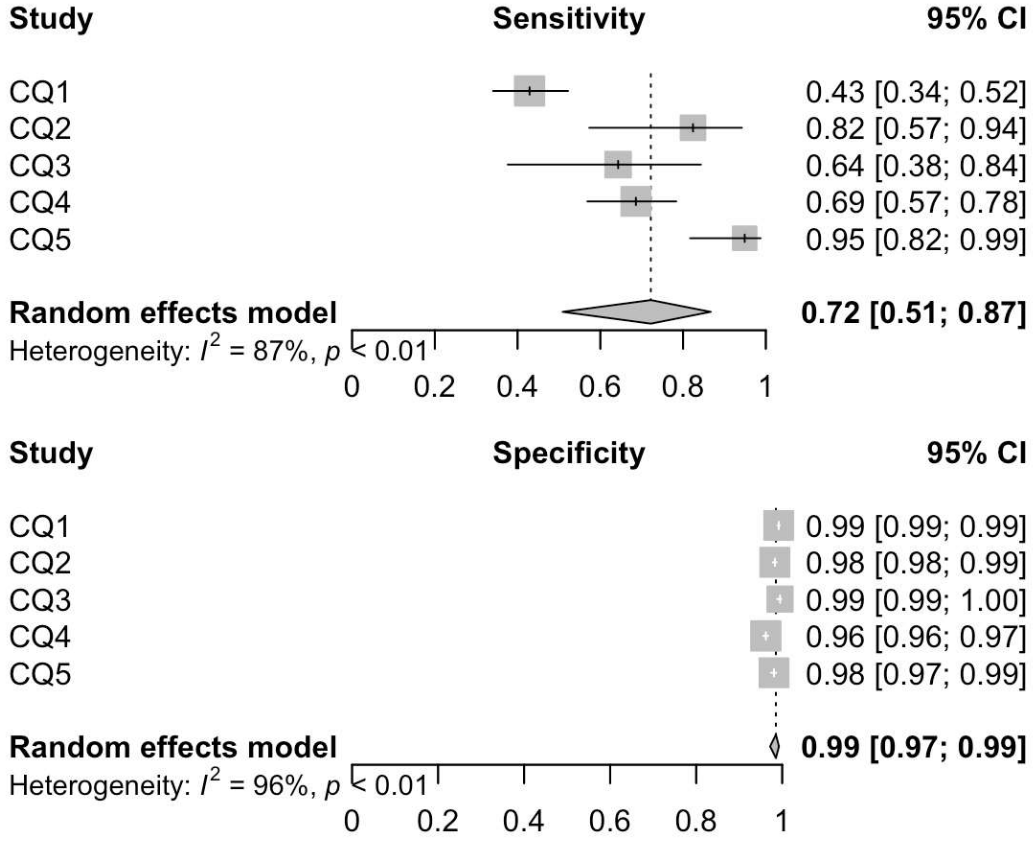

Post hoc secondary analysis adopted the modified prompt and the chain of thought strategy. The results of the included publications for the full text screening session using the conventional method were used as the standard reference. In the upper panel, the individual sensitivity for each clinical question (CQ) and integrated sensitivities across CQ1-5 are shown, with confidence intervals and inconsistency values (I^2). In the lower panel, the individual specificity for each CQ and integrated specificities across CQ1-5 are shown, with confidence intervals and inconsistency values (I^2).

© 2024 Oami T et al. *JAMA Network Open.*

eFigure 13. Forest Plots of Pairwise Meta-Analyses for Short-Term Mortality

$ScvO_2$ vs Usual care (full studies)

Study or Subgroup	$ScvO_2$ Events	$ScvO_2$ Total	Usual care Events	Usual care Total	Weight	Risk Ratio M-H, Random, 95% CI	Year
Gattinoni 1995	134	257	122	252	16.9%	1.08 [0.91, 1.28]	1995
Rivers 2001	38	130	59	133	11.3%	0.66 [0.47, 0.91]	2001
Wang 2006	4	16	7	17	2.2%	0.61 [0.22, 1.69]	2006
Chen 2007	26	88	50	102	9.8%	0.60 [0.41, 0.88]	2007
Jing 2010	39	157	62	146	11.2%	0.58 [0.42, 0.81]	2010
Peake 2014	147	792	150	796	15.7%	0.98 [0.80, 1.21]	2014
Yearly 2014	129	405	139	412	16.0%	0.94 [0.78, 1.15]	2014
Mouncey 2015	184	625	181	626	16.9%	1.02 [0.86, 1.21]	2015
**Total (95% CI)**		**2470**		**2484**	**100.0%**	**0.85 [0.72, 1.00]**	
Total events	701		770				

Heterogeneity: $Tau^2 = 0.03$; $Chi^2 = 21.61$, df = 7 (P = 0.003); $I^2 = 68\%$
Test for overall effect: Z = 1.98 (P = 0.05)

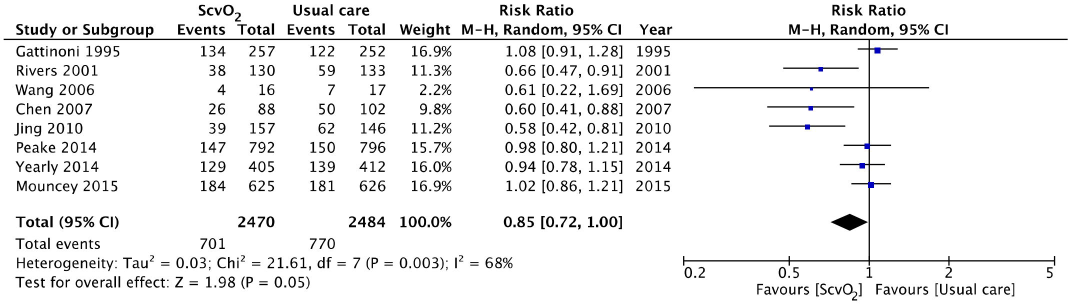

$ScvO_2$ vs Usual care (two studies excluded [Chen 2007, Gattinoni 1995])

Study or Subgroup	$ScvO_2$ Events	$ScvO_2$ Total	Usual care Events	Usual care Total	Weight	Risk Ratio M-H, Random, 95% CI	Year
Rivers 2001	38	130	59	133	15.0%	0.66 [0.47, 0.91]	2001
Wang 2006	4	16	7	17	2.8%	0.61 [0.22, 1.69]	2006
Jing 2010	39	157	62	146	14.9%	0.58 [0.42, 0.81]	2010
Yearly 2014	129	405	139	412	22.1%	0.94 [0.78, 1.15]	2014
Peake 2014	147	792	150	796	21.6%	0.98 [0.80, 1.21]	2014
Mouncey 2015	184	625	181	626	23.5%	1.02 [0.86, 1.21]	2015
**Total (95% CI)**		**2125**		**2130**	**100.0%**	**0.84 [0.71, 1.01]**	
Total events	541		598				

Heterogeneity: $Tau^2 = 0.03$; $Chi^2 = 13.47$, df = 5 (P = 0.02); $I^2 = 63\%$
Test for overall effect: Z = 1.83 (P = 0.07)

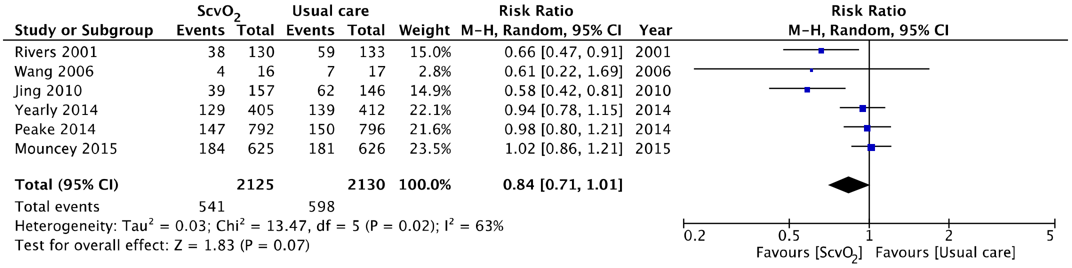

Lactate vs $ScvO_2$ (full studies)

Study or Subgroup	Lactate Events	Lactate Total	$ScvO_2$ Events	$ScvO_2$ Total	Weight	Risk Ratio M-H, Random, 95% CI	Year
Jones 2010	25	150	34	150	29.8%	0.74 [0.46, 1.17]	2010
Tian 2012	14	43	12	19	21.2%	0.52 [0.30, 0.89]	2012
Yu 2013	5	25	7	25	6.4%	0.71 [0.26, 1.95]	2013
Zhou 2017	33	180	50	180	42.6%	0.66 [0.45, 0.97]	2017
**Total (95% CI)**		**398**		**374**	**100.0%**	**0.65 [0.50, 0.84]**	
Total events	77		103				

Heterogeneity: $Tau^2 = 0.00$; $Chi^2 = 1.02$, df = 3 (P = 0.80); $I^2 = 0\%$
Test for overall effect: Z = 3.33 (P = 0.0009)

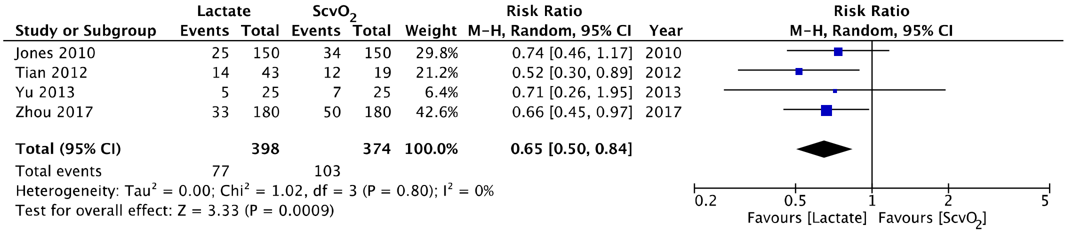

Lactate vs $ScvO_2$ (one study excluded [Tian 2012])

Study or Subgroup	Lactate Events	Lactate Total	$ScvO_2$ Events	$ScvO_2$ Total	Weight	Risk Ratio M-H, Random, 95% CI	Year
Jones 2010	25	150	34	150	37.8%	0.74 [0.46, 1.17]	2010
Yu 2013	5	25	7	25	8.1%	0.71 [0.26, 1.95]	2013
Zhou 2017	33	180	50	180	54.1%	0.66 [0.45, 0.97]	2017
**Total (95% CI)**		**355**		**355**	**100.0%**	**0.69 [0.52, 0.92]**	
Total events	63		91				

Heterogeneity: $Tau^2 = 0.00$; $Chi^2 = 0.13$, df = 2 (P = 0.94); $I^2 = 0\%$
Test for overall effect: Z = 2.53 (P = 0.01)

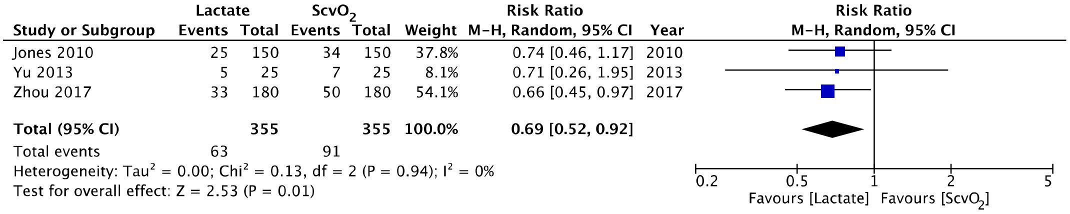

No excluded studies for the comparison between CRT vs Lactate and $P(v\text{-}a)CO_2/C(a\text{-}v)O_2$ vs $ScvO_2$.

* The data for this meta-analysis were extracted from the published systematic review for CQ4.[4] Forest plots of pairwise meta-analyses for full studies are re-created based on the extracted data. Two studies (Chen 2007, Gattinoni 1995) are judged as false-negative in the LLM assisted screening. Thus, we show the forest plot excluding these two studies if these studies are included in the meta-analysis.

© 2024 Oami T et al. *JAMA Network Open.*

eFigure 14. Forest Plots of Pairwise Meta-Analyses for ICU Mortality

$ScvO_2$ vs Usual care (full studies)

Study or Subgroup	$ScvO_2$ Events	$ScvO_2$ Total	Usual care Events	Usual care Total	Weight	Risk Ratio M-H, Random, 95% CI	Year
Gattinoni 1995	134	257	122	252	39.5%	1.08 [0.91, 1.28]	1995
Chen 2007	26	88	50	102	27.6%	0.60 [0.41, 0.88]	2007
Peake 2014	79	725	85	661	32.9%	0.85 [0.64, 1.13]	2014
**Total (95% CI)**		**1070**		**1015**	**100.0%**	**0.85 [0.61, 1.17]**	
Total events	239		257				

Heterogeneity: $Tau^2 = 0.06$; $Chi^2 = 8.38$, df = 2 (P = 0.02); $I^2 = 76\%$
Test for overall effect: Z = 1.00 (P = 0.32)

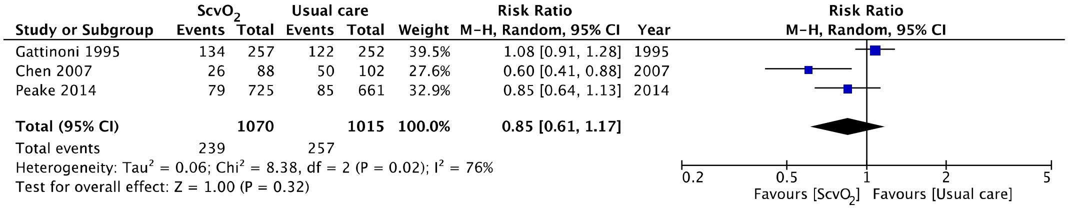

$ScvO_2$ vs Usual care (two studies excluded [Chen 2007, Gattinoni 1995])

Study or Subgroup	$ScvO_2$ Events	$ScvO_2$ Total	Usual care Events	Usual care Total	Weight	Risk Ratio M-H, Random, 95% CI	Year
Peake 2014	79	725	85	661	100.0%	0.85 [0.64, 1.13]	2014
**Total (95% CI)**		**725**		**661**	**100.0%**	**0.85 [0.64, 1.13]**	
Total events	79		85				

Heterogeneity: Not applicable
Test for overall effect: Z = 1.13 (P = 0.26)

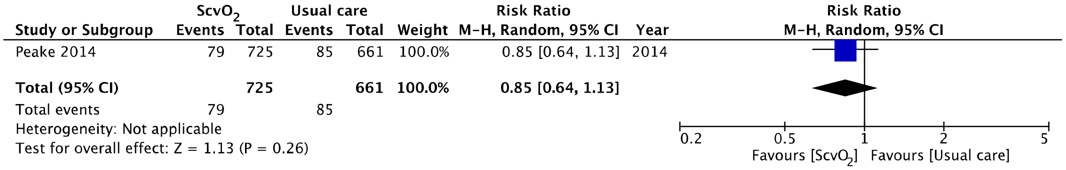

No excluded studies for the comparison between Lactate vs Usual care.

* The data for this meta-analysis were extracted from the published systematic review for CQ4.[4] Forest plots of pairwise meta-analyses for full studies are re-created based on the extracted data. Two studies (Chen 2007, Gattinoni 1995) are judged as false-negative in the LLM assisted screening. Thus, we show the forest plot excluding these two studies if these studies are included in the meta-analysis.

© 2024 Oami T et al. *JAMA Network Open.*

eFigure 15. Forest Plots of Pairwise Meta-Analyses for ICU Length Of Stay

$ScvO_2$ vs Usual care (full studies)

Study or Subgroup	$ScvO_2$ Mean	$ScvO_2$ SD	$ScvO_2$ Total	Usual care Mean	Usual care SD	Usual care Total	Weight	Mean Difference IV, Random, 95% CI	Year
Gattinoni 1995	24	27	257	26	50	252	0.6%	−2.00 [−9.00, 5.00]	1995
Jing 2010	19.9	2.2	157	20.6	1.9	146	25.7%	−0.70 [−1.16, −0.24]	2010
Yearly 2014	5.1	6.3	401	4.7	5.8	393	18.3%	0.40 [−0.44, 1.24]	2014
Peake 2014	3.1	2.8	792	3.3	3.1	796	28.8%	−0.20 [−0.49, 0.09]	2014
Mouncey 2015	3.1	3.6	625	2.5	3.9	626	26.6%	0.60 [0.18, 1.02]	2015
**Total (95% CI)**			**2232**			**2213**	**100.0%**	**−0.02 [−0.58, 0.54]**	

Heterogeneity: $Tau^2 = 0.26$; $Chi^2 = 19.37$, df = 4 (P = 0.0007); $I^2 = 79\%$
Test for overall effect: Z = 0.06 (P = 0.95)

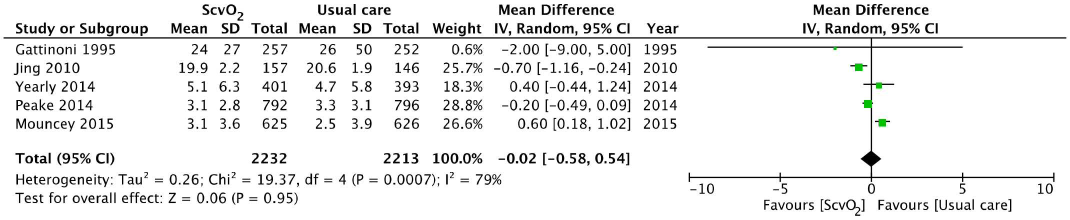

$ScvO_2$ vs Usual care (one study excluded [Gattinoni 1995])

Study or Subgroup	$ScvO_2$ Mean	$ScvO_2$ SD	$ScvO_2$ Total	Usual care Mean	Usual care SD	Usual care Total	Weight	Mean Difference IV, Random, 95% CI	Year
Jing 2010	19.9	2.2	157	20.6	1.9	146	25.9%	−0.70 [−1.16, −0.24]	2010
Peake 2014	3.1	2.8	792	3.3	3.1	796	28.8%	−0.20 [−0.49, 0.09]	2014
Yearly 2014	5.1	6.3	401	4.7	5.8	393	18.6%	0.40 [−0.44, 1.24]	2014
Mouncey 2015	3.1	3.6	625	2.5	3.9	626	26.7%	0.60 [0.18, 1.02]	2015
**Total (95% CI)**			**1975**			**1961**	**100.0%**	**−0.00 [−0.57, 0.57]**	

Heterogeneity: $Tau^2 = 0.27$; $Chi^2 = 19.08$, df = 3 (P = 0.0003); $I^2 = 84\%$
Test for overall effect: Z = 0.01 (P = 0.99)

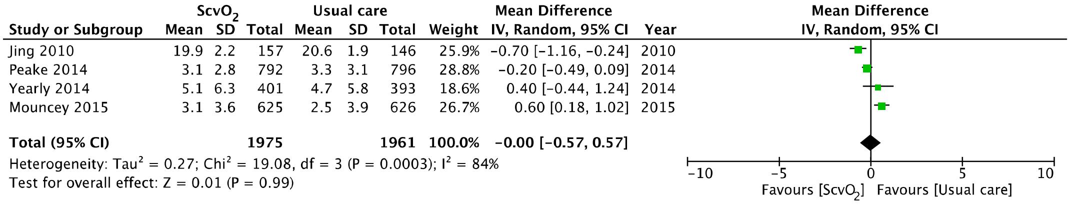

Lactate vs $ScvO_2$ (full studies)

Study or Subgroup	Lactate Mean	Lactate SD	Lactate Total	$ScvO_2$ Mean	$ScvO_2$ SD	$ScvO_2$ Total	Weight	Mean Difference IV, Random, 95% CI	Year
Jones 2010	5.9	8.5	150	5.6	7.4	150	24.3%	0.30 [−1.50, 2.10]	2010
Tian 2012	7.9	6	22	11.3	6	19	8.5%	−3.40 [−7.08, 0.28]	2012
Yu 2013	8	3	25	10	4	25	22.0%	−2.00 [−3.96, −0.04]	2013
Zhou 2017	3.2	3.6	180	3.6	4.3	180	45.2%	−0.40 [−1.22, 0.42]	2017
**Total (95% CI)**			**377**			**374**	**100.0%**	**−0.84 [−2.00, 0.33]**	

Heterogeneity: $Tau^2 = 0.61$; $Chi^2 = 5.37$, df = 3 (P = 0.15); $I^2 = 44\%$
Test for overall effect: Z = 1.41 (P = 0.16)

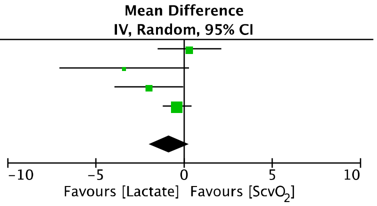

Lactate vs $ScvO_2$ (one study excluded [Tian 2012])

Study or Subgroup	Lactate Mean	Lactate SD	Lactate Total	$ScvO_2$ Mean	$ScvO_2$ SD	$ScvO_2$ Total	Weight	Mean Difference IV, Random, 95% CI	Year
Jones 2010	5.9	8.5	150	5.6	7.4	150	23.4%	0.30 [−1.50, 2.10]	2010
Yu 2013	8	3	25	10	4	25	20.6%	−2.00 [−3.96, −0.04]	2013
Zhou 2017	3.2	3.6	180	3.6	4.3	180	56.0%	−0.40 [−1.22, 0.42]	2017
**Total (95% CI)**			**355**			**355**	**100.0%**	**−0.57 [−1.58, 0.45]**	

Heterogeneity: $Tau^2 = 0.31$; $Chi^2 = 3.06$, df = 2 (P = 0.22); $I^2 = 35\%$
Test for overall effect: Z = 1.09 (P = 0.28)

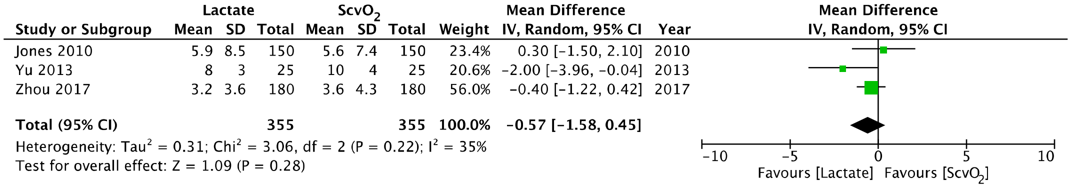

No excluded studies for the comparison between Lactate vs Usual care, CRT vs Lactate, and $P(v\text{-}a)CO_2/C(a\text{-}v)O_2$ vs $ScvO_2$.

* The data for this meta-analysis were extracted from the published systematic review for CQ4.[4] Forest plots of pairwise meta-analyses for full studies are re-created based on the extracted data. Two studies (Chen 2007, Gattinoni 1995) are judged as false-negative in the LLM assisted screening. Thus, we show the forest plot excluding these two studies if these studies are included in the meta-analysis.

© 2024 Oami T et al. *JAMA Network Open.*

eFigure 16. Forest Plots of Pairwise Meta-Analyses for Ventilator-Free Days

$ScvO_2$ vs Usual care (full studies)

Study or Subgroup	$ScvO_2$ Mean	SD	Total	Usual care Mean	SD	Total	Weight	Mean Difference IV, Random, 95% CI	Year
Mouncey 2015	19.6	12.1	625	19.8	12	626	100.0%	-0.20 [-1.54, 1.14]	2015
**Total (95% CI)**			**625**			**626**	**100.0%**	**-0.20 [-1.54, 1.14]**	

Heterogeneity: Not applicable
Test for overall effect: Z = 0.29 (P = 0.77)

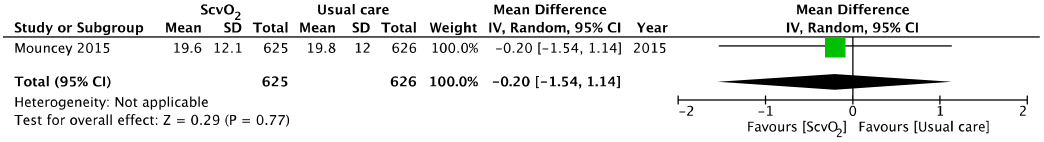

Lactate vs $ScvO_2$ (full studies)

Study or Subgroup	Lactate Mean	SD	Total	$ScvO_2$ Mean	SD	Total	Weight	Mean Difference IV, Random, 95% CI	Year
Jones 2010	9.3	10.3	150	9.9	11.1	150	100.0%	-0.60 [-3.02, 1.82]	2010
**Total (95% CI)**			**150**			**150**	**100.0%**	**-0.60 [-3.02, 1.82]**	

Heterogeneity: Not applicable
Test for overall effect: Z = 0.49 (P = 0.63)

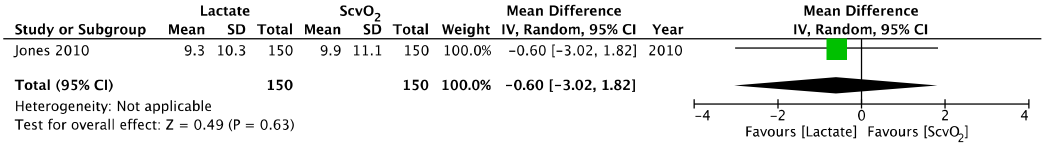

CRT vs Lactate (full studies)

Study or Subgroup	CRT Mean	SD	Total	Lactate Mean	SD	Total	Weight	Mean Difference IV, Random, 95% CI	Year
Hernández 2019	14.6	12.1	212	12.7	12.2	212	100.0%	1.90 [-0.41, 4.21]	2019
**Total (95% CI)**			**212**			**212**	**100.0%**	**1.90 [-0.41, 4.21]**	

Heterogeneity: Not applicable
Test for overall effect: Z = 1.61 (P = 0.11)

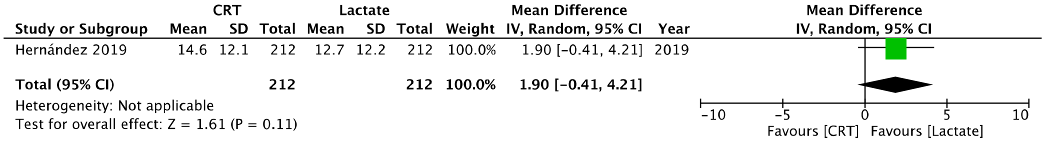

$P(v\text{-}a)CO_2/C(a\text{-}v)O_2$ vs $ScvO_2$

Study or Subgroup	$P(v\text{-}a)CO_2/C(a\text{-}v)O_2$ Mean	SD	Total	$ScvO_2$ Mean	SD	Total	Weight	Mean Difference IV, Random, 95% CI	Year
Su 2018	18.3	13.5	114	21.7	7.5	114	100.0%	-3.40 [-6.23, -0.57]	2018
**Total (95% CI)**			**114**			**114**	**100.0%**	**-3.40 [-6.23, -0.57]**	

Heterogeneity: Not applicable
Test for overall effect: Z = 2.35 (P = 0.02)

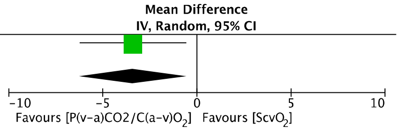

No excluded studies for the comparison between $ScvO_2$ vs Usual care, Lactate vs $ScvO_2$, CRT vs Lactate, and $P(v\text{-}a)CO_2/C(a\text{-}v)O_2$ vs $ScvO_2$.

*The data for this meta-analysis were extracted from the published systematic review for CQ4.[4] Forest plots of pairwise meta-analyses for full studies are re-created based on the extracted data. Two studies (Chen 2007, Gattinoni 1995) are judged as false-negative in the LLM assisted screening. Thus, we show the forest plot excluding these two studies if these studies are included in the meta-analysis.

© 2024 Oami T et al. *JAMA Network Open.*

eReferences

1. Gotschall T. EndNote 20 desktop version. *J Med Libr Assoc*. Jul 1 2021;109(3):520-522. doi:10.5195/jmla.2021.1260
2. Ouzzani M, Hammady H, Fedorowicz Z, Elmagarmid A. Rayyan-a web and mobile app for systematic reviews. *Syst Rev*. Dec 5 2016;5(1):210. doi:10.1186/s13643-016-0384-4
3. Oami T, Okada Y, Sakuraya M, Fukuda T, Shime N, Nakada TA. Efficiency and workload reduction of semi-automated citation screening software for creating clinical practice guidelines: a prospective observational study. *J Epidemiol*. Dec 16 2023;doi:10.2188/jea.JE20230227
4. Yumoto T, Kuribara T, Yamada K, et al. Clinical parameter-guided initial resuscitation in adult patients with septic shock: A systematic review and network meta-analysis. *Acute Med Surg*. Jan-Dec 2023;10(1):e914. doi:10.1002/ams2.914

© 2024 Oami T et al. *JAMA Network Open.*
