# Supplementary material for: Performance of a Large Language Model in Screening Citations
Source: JAMA Netw Open. 2024 Jul 8;7(7):e2420496. doi: 10.1001/jamanetworkopen.2024.20496 (PMC11231796; doi:10.1001/jamanetworkopen.2024.20496)
Supplement: Supplement 2. — Data Sharing Statement [file jamanetwopen-e2420496-s002.pdf]

Data Sharing Statement

Oami. Performance of a Large Language Model in Screening Citations. *JAMA Netw Open*. Published July 08, 2024. doi:10.1001/jamanetworkopen.2024.20496

Data
Data available: Yes
Data types: Data (not involving human participants)
How to access data: The datasets used and analyzed in this study are available from the corresponding author upon reasonable request.
When available: With publication

Supporting Documents
Document types: Statistical/analytic code
How to access documents: The code for this process is available at https://github.com/seveneleven711thanks39/gpt-assisted_citation_screening.git.
When available: beginning date: 02-16-2024

Additional Information
Who can access the data: Anyone requesting the data
Types of analyses: For any purpose
Mechanisms of data availability: With investigator support
